# Revealing the transfer pathways of cyanobacterial-fixed N into the boreal forest through the feather-moss microbiome

**DOI:** 10.3389/fpls.2022.1036258

**Published:** 2022-12-09

**Authors:** María Arróniz-Crespo, Jeremy Bougoure, Daniel V. Murphy, Nick A. Cutler, Virginia Souza-Egipsy, Dominique L. Chaput, Davey L. Jones, Nicholas Ostle, Stephen C. Wade, Peta L. Clode, Thomas H. DeLuca

**Affiliations:** ^1^ School of Natural Sciences, Bangor University, Bangor, United Kingdom; ^2^ School of Agricultural Engineering, CEIGRAM, Universidad Politecnica de Madrid, Madrid, Spain; ^3^ School of Agriculture and Environment, The University of Western Australia, Perth, WA, Australia; ^4^ Centre for Microscopy, Characterisation and Analysis, The University of Western Australia, Perth, WA, Australia; ^5^ Centre for Sustainable Farming Systems, Food Futures Institute, Murdoch University, Murdoch, WA, Australia; ^6^ Department of Geography, Scott Polar Research Institute, Cambridge, United Kingdom; ^7^ School of Geography, Politics and Sociology, Newcastle University, Newcastle, United Kingdom; ^8^ Servicio de Microscopıa Electronica, Instituto Ciencias Agrarias CSIC, Madrid, Spain; ^9^ Biosciences, University of Exeter, Exeter, United Kingdom; ^10^ Lancaster Environment Centre, Lancaster University, Lancaster, United Kingdom; ^11^ Advanced Microscopy and Bioimaging, Institute of Biological, Environmental and Rural Sciences, Aberystwyth University, Aberystwyth, United Kingdom; ^12^ School of Biological Sciences, The University of Western Australia, Perth, WA, Australia; ^13^ Department of Forest Ecosystems & Society, College of Forestry, Oregon State University, Corvallis, OR, United States

**Keywords:** biological N_2_ fixation, boreal forest, moss-cyanobacteria associations, moss microbiome, NanoSIMS, nitrogen cycling, *Pleurozium schreberi*

## Abstract

**Introduction:**

Biological N_2_ fixation in feather-mosses is one of the largest inputs of new nitrogen (N) to boreal forest ecosystems; however, revealing the fate of newly fixed N within the bryosphere (i.e. bryophytes and their associated organisms) remains uncertain.

**Methods:**

Herein, we combined ^15^N tracers, high resolution secondary ion mass-spectrometry (NanoSIMS) and a molecular survey of bacterial, fungal and diazotrophic communities, to determine the origin and transfer pathways of newly fixed N_2_ within feather-moss (*Pleurozium schreberi*) and its associated microbiome.

**Results:**

NanoSIMS images reveal that newly fixed N_2_, derived from cyanobacteria, is incorporated into moss tissues and associated bacteria, fungi and micro-algae.

**Discussion:**

These images demonstrate that previous assumptions that newly fixed N_2_ is sequestered into moss tissue and only released by decomposition are not correct. We provide the first empirical evidence of new pathways for N_2_ fixed in feather-mosses to enter the boreal forest ecosystem (i.e. through its microbiome) and discuss the implications for wider ecosystem function.

## Introduction

Cyanobacterial N_2_ fixation within feather-moss communities is a primary source of new nitrogen (N) in boreal forest ecosystems (DeLuca et al., 2002a, DeLuca et al., 2002b; Zackrisson et al., 2004; DeLuca et al., 2008; Renaudin et al., 2022a, Renaudin et al., 2022b); however, understanding of how this N source influences key ecosystem processes (e.g. productivity, biodiversity and carbon [C] cycling) remains limited with the precise cellular mechanisms yet to be defined. This is exemplified by the lack of knowledge about the fate of fixed N and its contribution to N nutrition of coexisting organisms (Jones and Wilson, 1978; Lindo et al., 2013; Kardol et al., 2016; DeLuca et al., 2022). Previous studies have demonstrated that N_2_ fixed within the moss-cyanobacteria system is highly conserved, with little direct transfer to higher plants and soil (Hyodo et al., 2013; Rousk et al., 2014; DeLuca et al., 2022). The lack of specialized symbiotic structures to facilitate transfer of N between cyanobacteria and its host moss (Solheim and Zielke, 2002) makes it difficult to assess how, and to what extent, nutrient exchange occurs between them. For example, direct uptake of fixed N has been reported in moss species such as *Sphagnum* (Berg et al., 2013), *Hymenostylium recurvirostrum* (Hedw.) Dixon (Jones and Wilson, 1978) and *Pleurozium schreberi* (Brid.) Mitt. (Bay et al., 2013). However, other studies have shown that not all the fixed N is directly transferred to *P. schreberi* (Kardol et al., 2016) or have failed to detect any direct transfer to moss tissue (Hyodo et al., 2013).

Even less is known about the relationship between the cyanobacteria and other components of the moss microbiome. All organisms within the boreal bryosphere require N for their maintenance, growth and reproduction, so competition between the moss and its associated-microbiome to access newly fixed N is likely. Further, competition can be expected to differ spatially based on vertical differentiation of both moss traits, e.g. physiological decline with ageing (Bates, 1979), and bryosphere microbial communities (Solheim and Zielke, 2002; Lindo and Gonzalez, 2010; Osono and Trofymow, 2012; Davey et al., 2013; Xiang et al., 2014). However, our understanding of fixed N dynamics within the bryosphere has traditionally only considered the moss and more specifically the presence and activity of cyanobacteria. More recently, some studies have demonstrated that mosses host a broad diversity of other putative N_2_-fixing bacterial lineages, for examples studies on *Sphagnum* mosses (Bragina et al., 2012; Bragina et al., 2013; Ho and Bodelier, 2015) and boreal mosses (Holland-Moritz et al., 2018). Consequently, uncertainty exists regarding both the pathways followed by cyanobacterial-fixed N within the moss-cyanobacteria system (Lindo et al., 2013; Ho and Bodelier, 2015; Kardol et al., 2016) and the possible role of other N_2_-fixers as N source in the boreal bryosphere.

Here we aim to describe the microbial and diazotrophic community associated with *P. schreberi* and trace the accumulation of newly fixed ^15^N_2_ in the moss and its associated microbiome to: 1) Identify the primary N_2_ fixing organisms in *P. schreberi*, a dominant moss species in the boreal forest; 2) Determine whether microbes living in association with the moss can access the fixed N; 3) Investigate if there is spatial differentiation for fixed N accumulation by moss cells related to tissue age (*i.e.*, between cells in the young tip of the moss stem and the old bottom segments); 4) Evaluate the potential implications of the results in the context of boreal ecosystem functioning.

The boreal forest is the largest terrestrial biome on earth and its ability to deliver a range of globally-important ecosystem services is critically dependent on the availability of N (DeLuca and Boisvenue, 2012). Our study offers evidence of rapid pathways of N fixed in feather-mosses into the boreal forest soil (i.e., through the moss microbiome rather than moss tissue decomposition), with implications for wider ecosystem function (i.e., a rapid route for N_2_ fixation to influence ecosystem functions compared with N release by decomposition of moss tissues).

## Material and methods

### Site description and ^15^N_2_ incubation experiment

This study was conducted in the boreal forest of Northern Sweden (65°46′-65°56′N, 18°20′E-19°6′E) where feather-moss carpets dominate the forest floor. The characteristics of the landscape in this area have been described in detail elsewhere (Zackrisson et al., 1996; DeLuca et al., 2002a; Zackrisson et al., 2004; DeLuca et al., 2022). We selected two forest sites with different canopy structure and nitrogenase activity (i.e., proxy of N_2_ fixation): Njällatjirelg, an open canopy forest with high forest floor moss N_2_ fixation and Reivo, a variably dense canopy forest with moderately high N_2_ fixation in the moss layer (**Supplementary Figure 1**). In September 2013, we conducted a ^15^N-labeled tracer addition experiment using ^15^N_2_ gas (**Supplementary Figure 2**). Three moss sample cores (including living and dead segments of the moss shoot, litter layer and humus soil) were collected at each forest site (Njällatjirelg and Reivo) using stainless steel cores (20 cm × 7 cm dia) and subsequently placed into acrylic tubes (20 cm × 7 cm dia) ensuring all cores had a headspace of 200 ml (extra humus soil was used to fill the bottom empty space when needed). Moss cores were hydrated by spraying distilled water to ensure adequate moisture for optimum physiological activation for the moss and its associated microbiome. Within 24 h of collection each tube was then hermetically sealed at the top and bottom. The lid was fitted with a rubber septum to facilitate injection of the ^15^N_2_ gas. A total of 200 ml of headspace was removed from each incubation vessel and was replaced by 200 ml of ^15^N_2_ gas (98 atom % ^15^N enriched, Sigma-Aldrich, UK). 80% of the cylinder was filled with the moss core (living and dead segments of the moss shoot, litter layer and humus soil) that contained trapped air allowing oxic conditions during the incubation. All incubation vessels were placed together into holes in the moss cushions directly in the field in a forest located closed to our laboratory base at Silvermuseet (Arjeplog, Sweden; 65°57’43”N, 18°17’57”E). Continuous ^15^N_2_ incubation took place for one week, the upper and lower caps were then removed, opened tubes with moss samples were then placed back into the holes and six moss shoot samples were collected immediately after the incubation ceased (0 wk: one week ^15^N_2_ exposure) and one and two weeks after the incubation ceased (1wk: 1 week ^15^N_2_ exposures plus 1 week with open tube; 2wk: 1 week ^15^N_2_ exposures plus 2 weeks with open tube). The 0 wk exposure served as a control for ^15^N exposure. The one week exposure provided ample time for diazatrophs to fix ^15^N_2_ through nitrogenase activity. The one week and two week field incubations allowed for potential transfer of ^15^N assimilated by diazatrophs to moss tissue or other organisms within the moss microbiome. Three control samples (6 moss shoots each) for each forest site were collected before the injection of ^15^N_2_ to determine the natural abundance of ^15^N within the cores.

### Selection of moss samples for NanoSIMS analysis

To establish the optimal samples for the NanoSIMS measurements, bulk-levels of ^15^N enrichment of bryosphere samples from each forest site (i.e., Njällatjirelg and Reivo) were analyzed along the incubation period using IRMS (Methods S1). We selected the highest bulk (IRMS) ^15^N enriched samples for high resolution NanoSIMS analysis. Bulk ^15^N natural abundance level was determined from control samples collected before ^15^N_2_ addition.

### Resin embedding and sectioning

In parallel to IRMS sampling, twenty-four moss shoots (one shoot per sample core and time-point) were fixed for resin embedding and sectioning. Immediately after each collection, fully hydrated moss shoots were fixed in 3% (v/v) glutaraldehyde in 0.1 M phosphate buffer, pH 7.4 for 4 h. After the primary fixation, samples were rinsed three times with phosphate buffer and one individual shoot carefully stored in 15 ml buffer. Samples were shipped to the Instituto Ciencias Agrarias (CSIC, Spain) for osmium tetroxide post-fixation, resin embedding, and sectioning (Methods S2). Shoots from the highest bulk (IRMS) ^15^N enriched samples were selected for further fixation. Four branches per selected shoot (two from the green and two from the brown parts, **Supplementary Figure 4**) were embedded for sectioning. Three sections were prepared from each embedded sample for optical (0.35 µm thick), transmission electron microscopy (TEM; 80 nm thick) and dual TEM - NanoSIMS (150 nm thick) observations. Three moss shoots (two from Njällatjirelg and one Reivo) from the unlabeled samples were used as controls for NanoSIMS analysis.

### NanoSIMS analysis and image processing

*In situ* isotopic mapping was performed at The University of Western Australia using a NanoSIMS 50 (Cameca, Gennevilliers, France), with a 16 keV Cs^+^ primary ion beam as described in Methods S3. Statistics from each region of interest (ROI: discrete groups of pixels that define a particular feature) were calculated. Following Berry et al. (2013), individual ROIs were considered significantly enriched in ^15^N if the mean value atom % ^15^N was above the 95^th^ percent confidence interval of unlabeled control ROIs from each particular component of the bryosphere and if the measurement error (2σ, Poisson) was smaller than the difference between the atom % of the labeled sample and the mean atom % of unlabeled control samples. To be confident that results were representative, 540 ROIs were analyzed in thirteen rastered sections across the green portion of the stem (*n* = 7 individual branches, **Supplementary Figure 4** and **Supplementary Figure 5**), 352 ROIs in ten sections across the brown portion (*n* = 5 individual branches, **Supplementary Figure 4** and **Supplementary Figure 5**) and 86 ROIs in five rastered sections for unlabeled samples from the green portion of the stem (*n* = 3 individual branches). Samples from the brown segments of *P. schreberi* from Njällatjirelg were removed from data analysis after NanoSIMS measurements since we could not detect ^15^N enrichment despite the presence of cyanobacteria.

### Microbial molecular analysis

In parallel with NanoSIMS sampling, six *P. schreberi* stems from Njällatjirelg (open canopy and high cyanobacteria colonization) and Reivo (variably dense canopy and moderately high cyanobacteria colonization) were collected from twelve sampling locations from each forest site. Stems were divided into light green (new growth tissue), dark green (mature tissue) and brown (senescent tissue) segments. These were pooled according to site and sampling location. For each site, stem segments of the same type from locations 1-6 and 7-12 were pooled, resulting in two pooled samples per site, per stem segment (twelve samples in total). DNA was extracted from pooled stem samples with the MoBio PowerSoil Kit according to the manufacturer’s instructions, quantified with the Qubit dsDNA HS Assay Kit on a Qubit 1.0 fluorometer (Life Technologies Ltd), and diluted to 0.5 ng/μL in 10 mM Tris pH 8.5.

Three targets were amplified for paired-end 300 bp sequencing on the Illumina MiSeq platform: Bacterial/archaeal 16S rRNA V4 with primers 515fB/806rB, fungal ITS1 with primers ITS1Fngs/ITS2 and *nif*H with primers IGK3/DVV. Primer sequences are listed in **Supplementary Table 1**. Library preparation and multiplexing were carried out using a 2-step PCR approach with the Nextera XT Index Kit (Methods S4). Bioinformatic pipelines for the three amplicon data sets are described in Methods S5. 16S rRNA amplicon data were processed with mothur 1.38 (Schloss et al., 2009; Kozich et al., 2013), and ITS1 amplicon data were processed with mothur and ITSx (Bengtsson-Palme et al., 2013). The *nif*H amplicon pipeline included steps in mothur, in the RDP functional gene pipeline (Fish et al., 2013), and in ARB (Ludwig et al., 2004), as well as incorporating the classification and regression trees model (Frank et al., 2016) for assigning *nif*H sequences to clusters and identifying paralogues. Fungal OTUs were also assigned to putative trophic mode using the FunGuild tool (Nguyen et al., 2016).

## Results

### Accumulation of newly fixed ^15^N_2_ in the moss and its associated microbiome

**Figure 1:**
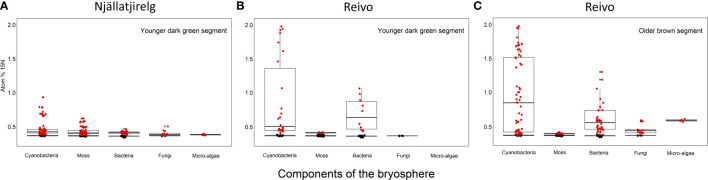
^15^N accumulation (atom% ^15^N) of metabolically active cell components of the bryosphere of two northern Sweden boreal forest after ^15^N_2_ incubation. **(A)** Upper younger dark green segment of *Pleurozium schreberi* shoot from Njällatjirelg, an open canopy forest with high moss N_2_ fixation, and **(B)** from Reivo, a variably dense canopy forest with moderately high N_2_ fixation in the moss layer, and **(C)** lower older brown segment of *P. schreberi* shoot from Reivo. Data from unlabeled replicates from each site used to generate ^15^N natural abundance values of each component - shown as black dots. Only data from the active (i.e. ^15^N enriched, red dots) cells is presented, > 95% confidence intervals of unlabeled controls and error (2σ, Poisson) smaller than the difference between the atom % of the labeled sample and the mean atom % of unlabeled control samples. Box plots summarize the quartiles of the target components of the bryosphere where boxes and whiskers encompass 25–75% and 5–95% quantiles of the data, respectively, with the median indicated by a dark horizontal line. Samples from the brown segments of *P. schreberi* from Njällatjirelg were removed from data analysis after NanoSIMS measurements since we could not detect ^15^N enrichment despite the presence of cyanobacteria. Branch location along the moss shoot selected for NanoSIMS analysis and raw images of rastered sections used for data analysis can be found in **Figure S4** and **Figure S6** respectively.

**Figure 2:**
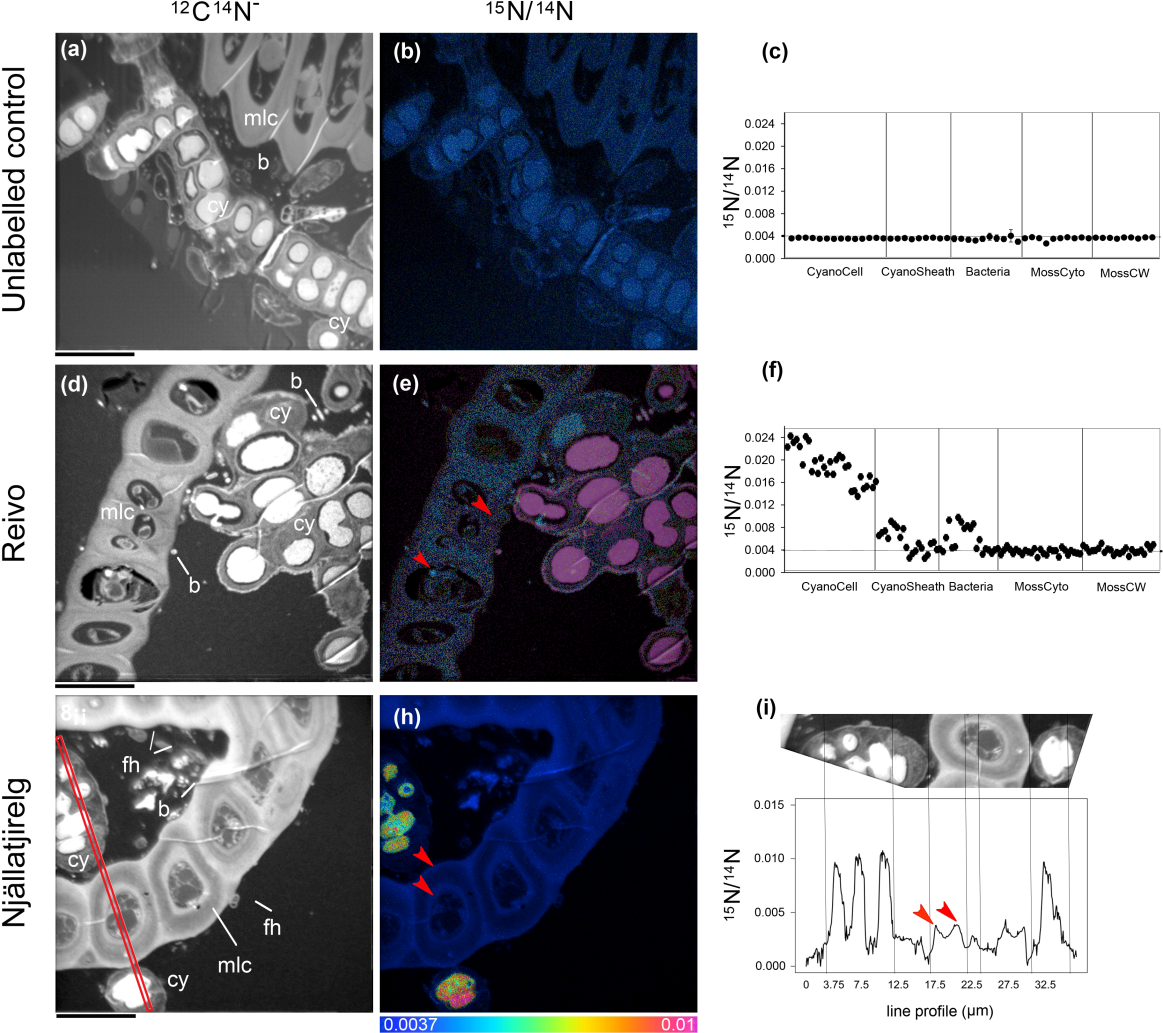
Spatial distribution of ^15^N accumulation in younger dark green segments of *Pleurozium schreberi* shoot and associated microbiome.^15^N concentration (^15^N/^14^N) of representative samples of an unlabeled shoot **(A–C)** and shoot labeled with ^15^N_2_ and incubated for one week (Reivo forest site d-f; Njällatjirelg forest site g-i). ^15^N enrichment can be distinguished in cyanobacteria cells (CyanoCell) and in the moss cell wall (MossCW) and cytoplasm (MossCyto) in both forest sites (f and red arrow in e, h and i for moss cells). Epiphytic bacterial cells in direct contact with cyanobacteria can be seen enriched in ^15^N **(D–F)**. Fungal hyphae attached to the moss leaf close to the cyanobacteria cells can be distinguished **(G)**. Cellular structures are visible in the greyscale ^12^C^14^N images **(A, D, G)**, with corresponding ^15^N/^14^N **(B, E, H)** images reflecting levels of ^15^N enrichment. The HSI colour scale (0.0037–0.01; ^15^N/^14^N natural abundance = 0.0037) applies to all HSI images. Dots and line scans show numerical levels of ^15^N enrichment (^15^N/^14^N) across the subcellular regions **(C, F, I)**, with data acquired from the line (512 × 512 pixels) **(I)** indicated on the respective ^12^C^14^N image **(G)**. Structural features: cyanobacteria (cy), bacteria (b), fungal hyphae (fh), and moss leaf cell (mlc). Bars, 10 µm (for all images).

**Figure 3:**
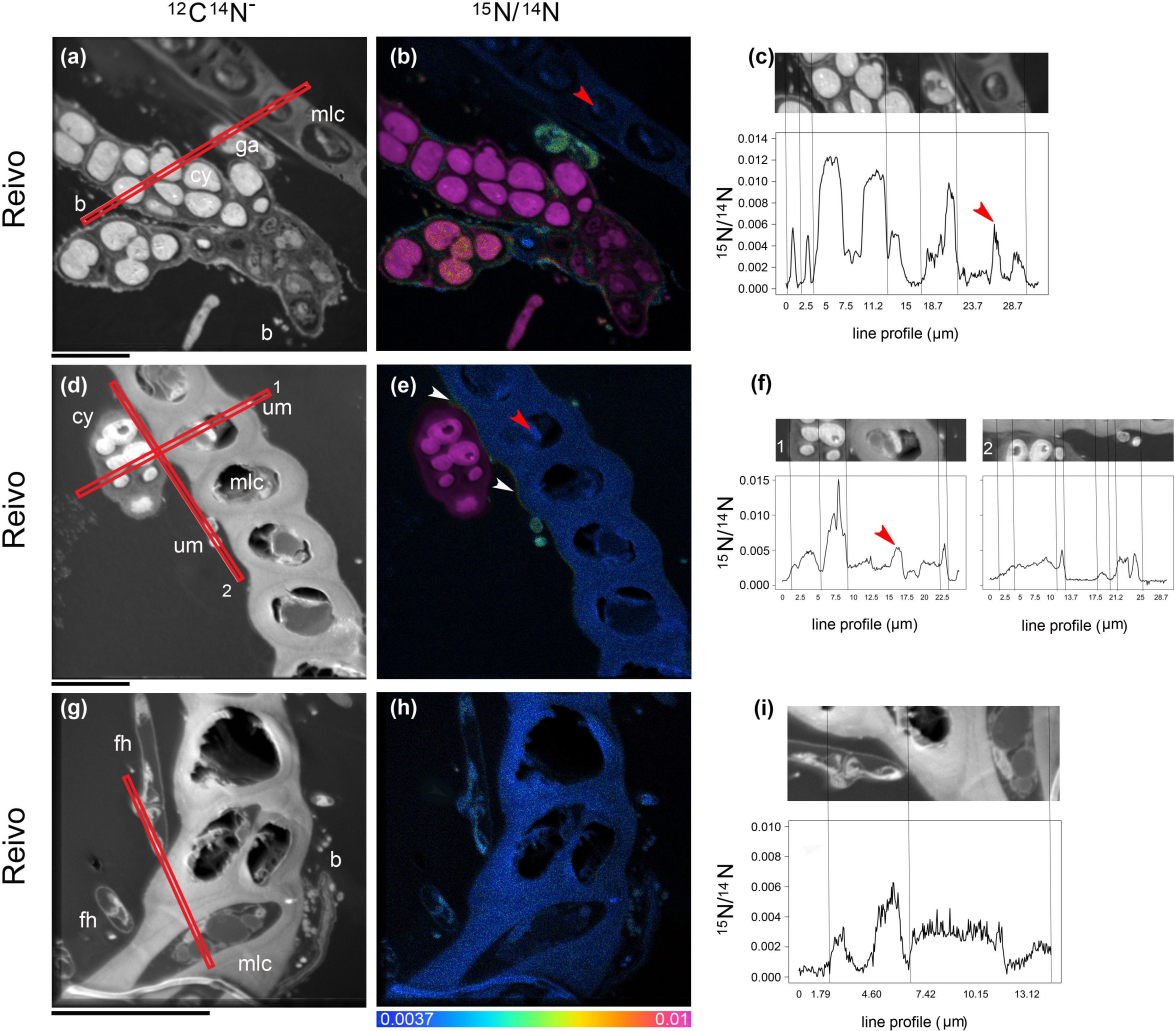
Spatial distribution of ^15^N accumulation in older brown segments of *Pleurozium schreberi* shoot and associated microbiome. Enrichment of both moss cells and moss-associated microbiome can be distinguished after one week of continuous exposure to ^15^N_2_ (a-i). Low ^15^N enrichment within the moss cell can be detected in some organelles (red arrow in **B, C** and **E, F**) whereas the cell wall and lipid bodies are not enriched (< 0.0037 ^15^N/^14^N, f). Epiphytic bacterial cells in direct contact with cyanobacteria can be seen enriched in ^15^N **(A–C)**; the uptake of ^15^N fixed by cyanobacteria cells into green algae **(A–C)** and a hypha from a Basidiomycete (distinguished by a clamp connection at the hyphal septa, white arrows in **G–I**) were also detected. In **(D–F)** an extracellular gradient of ^15^N enrichment is visible over the moss cell (indicated by two white arrows). Epiphytic microbes seated on the moss leaf have access to the fixed ^15^N and were enriched **(E, F)**, whereas the moss cell walls are not enriched (< 0.0037 ^15^N/^14^N). Cellular structures are visible in the greyscale ^12^C^14^N images **(A, D, G),** with corresponding ^15^N/^14^N **(B, E, H)** images reflecting levels of ^15^N enrichment. The HSI color scale (0.0037–0.01; ^15^N/^14^N natural abundance = 0.0037) applies to all HIS images. Line scans (512 x 512 pixels) show numerical levels of ^15^N enrichment (^15^N/^14^N) across the subcellular regions **(C, F, I)**, with data acquired from the lines indicated on each of the respective ^12^C^14^N images. Structural features: cyanobacteria (cy), bacteria (b), fungal hyphae (fh), unicellular green algae (ga), unidentified microbes (um) and moss leaf cell (mlc). Bars, 10 µm (for all images).

To trace the fate of fixed ^15^N_2_ at a cellular level, we correlated high-resolution morphology images (transmission electron microscopy and ^12^C^14^N NanoSIMS images) to sub-cellular scale isotope enrichment (> 0.37 atom % ^15^N) ^12^C^15^N NanoSIMS images (**Supplementary Figure 5**), to allow the identification of cell types by specific ultrastructure and the level of ^15^N enrichment accumulated. We detected ^15^N enriched cyanobacteria cells (i.e. metabolically active cells) associated with both the younger dark green segments of *P. schreberi* shoots (atom % ^15^N data = 0.47 and 0.53 [median] from Njällatjirelg and Reivo, respectively **Figures 1A, B**), and older brown segments (atom % ^15^N data = 0.85 [median] from Reivo, **Figure 1C**). In addition to cyanobacterial cells, ^15^N enrichment was observed in moss cells but the level of enrichment differed based on the age (proxy for metabolic activity; Bates, 1979) of moss tissues. In this sense, younger dark green moss segments showed higher ^15^N enrichment (moss atom % ^15^N data = 0.43 and 0.41 [median] from Njällatjirelg and Reivo respectively; range for both forests = 0.40 – 0.44, **Figures 1A, B**) than older brown moss segments (moss atom % ^15^N data = 0.39 [median] from Reivo; range = 0.39 – 0.40, **Figure 1C**). The percentage of ^15^N enriched regions of interest (ROIs, red dots in **Figure 1**), from the total analyzed, was also higher in the younger dark green moss segments (29%, *n* = 205) than in the older brown moss segments (18% *n* = 140). Specifically, ^15^N enrichment was located in the cell walls and cytoplasm of younger dark green segments of moss shoots (**Figures 2E, F**, from Reivo and 2h,i from Njällatjirelg: see red arrows) whereas in older brown moss segments, only discrete hotspots of ^15^N enrichment were observed in moss cell cytoplasm (**Figures 3B, C, E, F** from Reivo: see red arrows). Importantly, there was an extracellular ^15^N enrichment (probably extracellular polysaccharide; EPS) on older moss cell (**Figure 3E**, see two white arrows) that was accessible to epiphytic microbes (blue green color), whereas moss cell walls were not ^15^N enriched (≤ 0.37 atom % ^15^N, dark blue color).

The non-cyanobacterial microbiome (*i.e*. other bacteria, fungi, and micro-algae) associated with *P. schreberi* was enriched in ^15^N compared with unlabeled controls (**Figure 1**). The enrichment (> 0.37 atom % ^15^N) in heterotrophic bacterial cells was higher in Reivo (0.43 - 1.06 atom % ^15^N in younger green segments and 0.37 - 1.30 atom % ^15^N in older brown moss segments, **Figures 1B, C**) than in Njällatjirelg (range 0.37 – 0.47 atom % ^15^N in green segments, **Figure 1A**). Fungal cells associated with younger dark green segments in Njällatjirelg had an enrichment above natural abundance of 0.38 atom % ^15^N (median) with a maximum of 0.50 atom % (**Figure 1A**); in Reivo fungal cells associated with older brown moss segments had an enrichment of 0.44 atom % ^15^N (median) with a maximum of 0.59 atom % (**Figure 1C**). The ^15^N enrichment of micro-algae was between 0.38 and 0.59 atom % ^15^N (median) from Njällatjirelg and Reivo respectively; the highest ^15^N enrichment measured in an individual micro-algae cell was 0.40 atom % in Njällatjirelg and 0.61 atom % in Reivo (**Figures 1A, C**). NanoSIMS images also revealed that the highest ^15^N enrichment in bacteria cells were observed in close proximity to the cyanobacteria (**Figures 2D–F** *vs.* 2g-i and **Figures 3A–C** *vs.* 3g-i, **Supplementary Figure 6** for all rastered sections). NanoSIMS images also revealed uptake of ^15^N fixed by cyanobacteria cells into green algae (**Figures 3A–C**) and the *hyphae of a Basidiomycete*, distinguished by a clamp connection at the hyphal septa (**Figures 3G–I**: white arrow).

### Bacterial diversity and N fixation capacity of the moss microbiome

**Figure 4:**
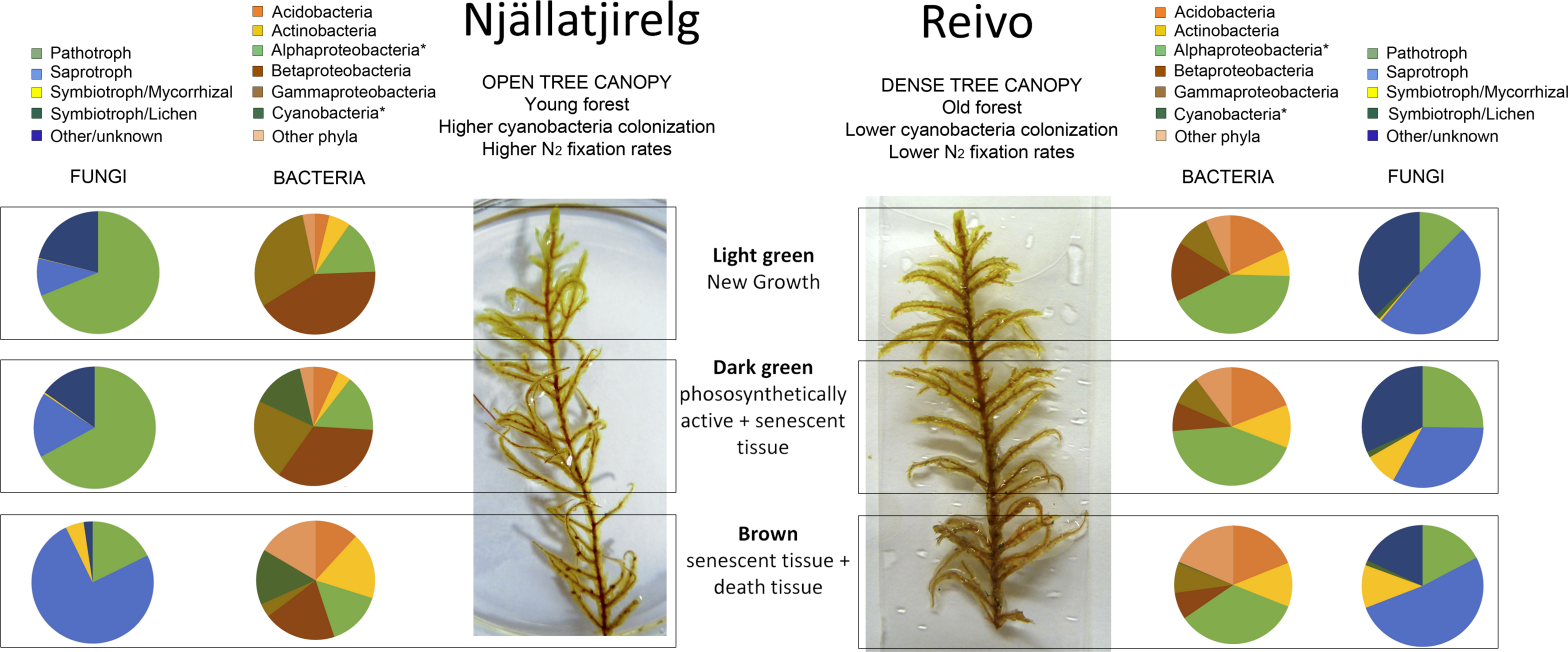
Bacterial and fungal community shifts along the *Pleurozium schreberi* shoot profile revealed by 16S and ITS analysis from two northern Sweden boreal forests. Pie charts represent relative abundance of the major bacterial taxa (brownish pie charts) and fungal trophic mode (blueish pie charts) associated with the light green (new growth tissue), dark green (mature tissue) and brown (senescent tissues) segments of the moss *P. schreberi*. Samples collected in Njällatjirelg, open canopy forest with high nitrogenase activity, and Reivo, variably dense canopy and moderately nitrogenase activity. Results are based on the number of sequence reads from taxa that could be assigned to genus level or lower (detailed results on **Supplementary Table 2** and **Supplementary Table 3**). Bacterial taxa marked with asterisks were present in the *nif*H data set (Cyanobacteria 99.90 % and Alphaproteobacteria 0.10 % of the total number of *nifH* sequences).

We estimated that ∼24% (*n* = 100) of the total moss-associated heterotrophic bacteria in Njällatjirelg and ~53% (*n* = 86) in Reivo were enriched in ^15^N (**Figure 1**, red dots), indicating active uptake of ^15^N_2_. To help elucidate whether these heterotrophic bacteria were capable of direct N_2_ fixation, we profiled both the total bacterial community (targeting the 16S rRNA gene) and the diazotrophic (N_2_ fixing) community (targeting the *nif*H gene that encodes nitrogenase subunits). The total bacterial communities across both forest sites were dominated by four phyla, namely Proteobacteria (classes Alpha-, Beta- and Gammaproteobacteria), Acidobacteria, Actinobacteria, and Cyanobacteria (**Figure 4** and **Supplementary Table 2**), with large relative abundances of potential N_2_ fixing proteobacterial taxa such as *Burkholderia* spp., *Pseudomonas* spp (Bragina et al., 2012; Bragina et al., 2013), and Alphaproteobacteria (Holland-Moritz et al., 2018).

In contrast, the *nif*H data were dominated by cyanobacterial sequences (**Supplementary Figure 7**), with heterotrophic bacterial *nif*H sequences, clustering with the Alphaproteobacteria, accounting for only 0.1% of the total (18 out of 17768 *nif*H sequences, **Supplementary Figure 7A**). The cyanobacterial *nif*H sequences fell into three clusters (**Supplementary Figure 7B**): i) a *Nostoc cluster* (0.60% total *nif*H reads), ii) a *Stigonema cluster* (0.15% total *nif*H reads) that has been described previously (Ininbergs et al., 2011) and contains *nif*H sequences of cyanobacteria found in association with *P. schreberi* and *Hylocomium splendens* (i.e. bands D, J, 6, 17 and clones A11, C11, E11) (Ininbergs et al., 2011), and iii) a third cluster, here provisionally named *Stigonema cluster II*, that included some sequences from cyanobacteria associated with boreal moss species (i.e. clones H10, G10, 6, 29 and 3-17) (Ininbergs et al., 2011; Leppanen et al., 2013), as well as numerous *Stigonema* species. *Stigonema cluster II* contained the majority of the *nif*H sequences generated in this study (99.15%), suggesting that this cluster is the main contributor of the newly fixed N input among N-fixer of the diazotrophic community studied here. The identification of these key community members was further confirmed by BLAST analyses of representative sequences from the 16S rRNA cyanobacterial OTUs, which showed that 97.2% of cyanobacterial 16S rRNA sequences could be placed in the genus *Stigonema*, with the remaining 2.8% of cyanobacterial sequences belonging to the genus *Nostoc*.

The 16S rRNA data showed distinct distribution profiles of bacterial OTUs between forest sites and tissue type (**Supplementary Figure 8**), with clear differences in relative abundance emerging at the phylum level (**Figure 4**). In particular, the distribution of cyanobacteria was not uniform, but rather was localized to the dark green and brown segments of *P. schreberi* from Njällatjirelg (∼14% both, **Figure 4**), with lower abundances detected in Reivo (∼0.34% brown segments, **Figure 4**), the lower N_2_ fixation site (**Supplementary Figure 1**). Betaproteobacteria and Gammaproteobacteria were more abundant in Njällatjirelg and on younger segments (light and dark green segments), notably *Burkholderia* spp. (21%), other Burkholderiales (15%) and *Pseudomonas* spp. (24%) (**Supplementary Table 1**). In Reivo, Alphaproteobacteria were more abundant (38%) - notably taxa from the order Rhizobiales (10%) and the Acetobacteraceae (16%) - and were evenly distributed along the moss shoot. Acidobacteria (18%), notably *Granulicella* spp., were more abundant on *P. schreberi* growing in Reivo and evenly distributed along the moss shoot (**Figure 4**). The relative abundance of Actinobacteria was similar between forest sites and increased with depth along the moss shoot (*i.e.*, higher abundance on old, brown segments at the base of stems) (**Figure 4**).

### Fungal diversity of the moss microbiome

Fungal community composition was strongly influenced by forest site and, to a lesser degree, by moss tissue type (**Supplementary Figure 8**). The bryosphere was dominated by fungi from the Ascomycota (74%); and Basidiomycota (21%) (**Supplementary Table 3**). Fungal community structure and composition varied between the forest sites. In Njällatjirelg, the fungal community was characterized by the saprotrophic fungi *Penicillium* sp., *Hypocrea* sp., *Cantharellula umbonata*, *Cystofilobasidium capitatum*, *Mortierella* sp., the parasitic fungi *Cystodendron* sp., *Phacidium lacerum*, the yeast *Cryptococcus victoriae* and the ericoid mycorrhizal fungi *Oidodendron* sp. At Reivo, the *P. schreberi-*associated fungal community was characterized by the parasitic fungi *Cadophora* sp., *Hyaloscypha* sp, *Venturia* sp., the saprotrophic fungi *Cladophialophora* sp. and *Rhodotorula* sp. and the ectomycorrhizal fungi *Clavulina* sp. Most taxa (161 genera, 52.4% of the total recovered sequences) could be confidently assigned a unique trophic mode (**Supplementary Table 4**). Plant pathogens were more abundant (∼ 68% of total assigned reads) in Njällatjirelg and associated with younger segments (light and dark green), while saprotrophs were more abundant in Reivo across the moss shoot and in particular associated with older brown segments (senescent tissues ∼50%, **Figure 4**). Symbiotrophic fungi were dominated by mycorrhizal fungi (**Figure 4**). Mycorrhizal fungi were mainly recovered from Reivo and associated with senescent tissue (dark green and brown segments, **Figure 4**, **Supplementary Table 5**).

## Discussion

Our results confirm that N newly fixed in feather-mosses can be readily transferred to moss cells and co-existing microbes. The idea that N_2_ fixed by cyanobacteria associated with mosses could be incorporated into other bryobiota was first suggested by Jones and Wilson (1978) four decades ago; however, since then, it has been raised by others, but never empirically addressed (Solheim et al., 2002; Solheim et al., 2004; Lindo et al., 2013; Kardol et al., 2016). Our results, obtained from two distinctive locations in the northern Swedish boreal forest, provide compelling evidence that not all of the N_2_ fixed in *P. schreberi* is transferred to the moss and at least some is consumed by organisms within the moss microbiome, which has implications for wider ecosystem function as discussed below. This new knowledge of fixed-N pathways is crucial to understanding the functional significance of moss-cyanobacteria associations in boreal forest ecosystems where N is limited.

### Fixed-N accumulation in multiple microbial groups within the boreal bryosphere

NanoSIMS images revealed the presence of ^15^N_2_ fixed within cyanobacteria cells and its presence adjacent moss cells and the moss microbiome, including heterotrophic bacteria, fungi and micro-algae. We propose three possible pathways for the transfer of fixed N, based on previous studies of Sphagnum-methanotroph systems (Ho and Bodelier, 2015) and bryophyte-cyanobacteria systems (Jones and Wilson, 1978; Lindo et al., 2013): 1) Fixed N may end up in the moss tissue and microbiome *via* an indirect pathway of cyanobacterial cell death and lysis; 2) Bacterivores grazing *P. schreberi* may ingest and subsequently release part of the N_2_ fixed by cyanobacteria prey cells; 3) A direct pathway involving an exchange of N-compounds between the cyanobacteria, moss tissue and moss microbiome. The close proximity of cyanobacteria, moss tissue and other microbial cells (*e.g.*, bacteria, micro-algae and fungi) coupled with the level of ^15^N enrichment observed on younger photosynthetically active moss cells (maximum 0.44 atom % ^15^N) and moss-microbiome (maximum 1.30, 0.59, 0.61 atom % ^15^N in heterotrophic bacterial, fungal and micro-algae cells) suggest a direct exchange of N; however, the first two pathways may also occur. On the other hand, the high level of ^15^N enrichment observed in some bacterial cells could also suggest the presence of other N_2_-fixers associated with the moss (e.g., N_2_ fixing proteobacteria) as discussed below.

The widespread incorporation of cyanobacteria-fixed ^15^N_2_ observed here has major ecological implications for N cycling and turnover within the broader boreal forest floor. Specifically, the capacity of the moss-cyanobacteria association to impact N cycling at ecosystem level depends on the fate of the newly fixed N within the bryosphere. Based on our observations, we confirm that fixed N can flow into the boreal ecosystem *via* a slow turnover pathway where N is incorporated into photosynthetically active moss tissues that are subsequently decomposed (Lang et al., 2009), and a fast pathway where N is incorporated into bacteria, fungal, and micro-algal biomass. In the former, the fixed N is highly conserved in the moss tissue (Turetsky, 2003; Gavazov et al., 2010; Rousk et al., 2014, Rousk et al., 2016), and slowly incorporated into the underlying litter and humus layer within a timescale of several years to decades (Lindo et al., 2013; DeLuca et al., 2022). In the latter, N_2_ fixed by cyanobacteria is rapidly available for transfer across trophic levels within the microbial and mesofaunal food web [e.g., bacterial food chain: bacteria, protozoa, rotifers, nematodes, arthropods, and the fungal food chain: saprophytic and mycorrhizal fungi, fungivorous arthropods, and nematodes (Lindo et al., 2013)]. These results are also consistent with the fast transfer of fixed ^15^N_2_ associated with mosses into other components of the biosphere (e.g. soil microbes, other plants) in a High Arctic, N-limited ecosystem (Rousk et al. (2017). These results support the view that cyanobacterial fixed-N can supplement the microbial food web associated with the mosses reinforcing the influence of this N source in key ecosystem processes in which bacterial- and fungal-based food webs are involved (i.e. organic matter decomposition, carbon sequestration, nutrient cycling; Moquin et al., 2012; Bragina et al., 2014; Jonsson et al., 2015; Kardol et al., 2016).

### Cyanobacterial N_2_ fixation is far greater than heterotrophic N_2_ fixation in mosses

The potential for heterotrophic N_2_ fixation has been suggested in feather-mosses (Warshan et al., 2016; Holland-Moritz et al., 2018); however, its relative contribution to the total N_2_ fixation in boreal forest is not clear. We observed that although heterotrophic *nif*H sequences were present, their relative abundance was low (0.1%, see **Supplementary Figure 7A**). Similar to previous research (Ininbergs et al., 2011; Leppanen et al., 2015; Warshan et al., 2016), our results indicate that cyanobacteria are the most abundant diazotrophic group, where others may also occur as casual epiphytes on the feather-moss. We detected an anomalously low number of cyanobacterial *nif*H reads from the Reivo forest, the mature forest site, despite cyanobacteria being readily observed under NanoSIMS and microscopy analysis (**Figures 2**, **3**). In particular, Warshan et al. (2016) showed variations of *nif*H gene abundance by sampling date, moss species and cyanobacterial cluster, which may partially explain differences in *nifH* abundance between both forest sites (*i.e.* this may reflect a primer bias due to cyanobacteria at Reivo being of a different strain or species from those at Njällatjirelg). In this light, we consider that even though the *nif*H gene has high taxonomic resolution among molecular markers used to study feather-moss associated cyanobacteria (as shown by Ininbergs et al., 2011), the actual number of cyanobacterial strains associated with boreal feathermosses may not necessarily be accurately estimated solely by using the *nif*H gene abundance (Gaby and Buckley, 2012; Warshan, 2017).

Given the caveats expressed above, the low contribution of heterotrophic N fixation was also apparent by linking taxonomic and functional patterns of Proteobacteria associated with *P. schreberi.* Despite the large relative abundances of putative N_2_ fixing proteobacterial taxa (Bragina et al., 2012; Bragina et al., 2013; Holland-Moritz et al., 2018) identified in our 16S rRNA assays in Reivo, we detected very few Proteobacterial *nif*H sequences (indicative of actual N_2_ fixation capacity) in both forest communities (only 10 sequences in Njällatjirelg samples and 8 sequences in Reivo: **Supplementary Figure 7A**). The high proportions of ^15^N-enriched bacteria found in both forest sites (24% in Njällatjirelg and 53% in Reivo) and, particularly, those found in close proximity to cyanobacteria (**Figures 2D–F** *vs.* 2g-i and **Figures 3A–C** *vs.* 3g-i, **Supplementary Figure 5** for all rastered sections) suggest, therefore, that N released from the cyanobacteria is more likely consumed by co-occurring heterotrophic bacteria than derived from N_2_ fixation by heterotrophic bacteria. To date, few studies have described the bacterial community structure in bryophyte communities with notable exception of *Sphagnum* species (Estrada-De los Santos et al., 2001; Opelt et al., 2007; Moquin et al., 2012; Bragina et al., 2013; Jassey et al., 2013; Bragina et al., 2014) and boreal feather-mosses (Ininbergs et al., 2011; Leppanen et al., 2015; Warshan et al., 2016; Holland-Moritz et al., 2018). Unlike previous studies, however, we directly link the bacterial community structure to boreal ecosystem functioning by combining high resolution isotope imaging (Nano-SIMS) and high-throughput amplicon sequencing of several targets (16S rRNA, *nif*H). Our results show that, most likely, cyanobacteria-fixed N is assimilated by heterotrophic bacteria; this may alleviate N-limitation in bacterial decomposer communities and flow up to other trophic levels within the bacterial food chain. Overall, our results demonstrate that bacteria are key players in the transformation of the cyanobacterial fixed-N within the boreal bryosphere, warranting further and more specific inquiry (*i.e.* to determine their growth dynamics, N loss pathways etc.).

### Fungal communities benefit from the newly fixed N_2_

The moss-associated fungal community detected here, represented a diverse assemblage of putative plant pathogens, saprotrophs and symbiotrophic fungi, which is consistent with previous reports of fungal community structure in boreal feather-mosses (Davey et al., 2017). NanoSIMS imaging results provide good evidence that fungi associated with *P. schreberi* do assimilate recently fixed N and into the fungal food chain consisting of saprophytic fungi, arbuscular- and ecto-mycorrhizal fungi, fungivorous arthropods, and nematodes (Lindo and Gonzalez, 2010). Remarkably, we found *hyphae from Basidiomycetes* enriched with ^15^N (**Figures 3G–I** white arrow) where mycorrhizae associated with the moss were more diverse and abundant (old segments of shoot samples from Reivo, **Figure 4**). Although these results do not prove a mycorrhizal pathway by which N fixed within the moss-cyanobacteria system is transferred to vascular plants (Carleton and Read, 1991; Lindo et al., 2013), it does provide new insight into the benefit obtained by the fungal community when growing in association within the bryosphere microbiome (Davey and Currah, 2006).

## Conclusions

During the past four decades, scientists have argued about the possible pathways by which N_2_ fixed by cyanobacteria associated with mosses is incorporated into the wider boreal biome. Our results provide, for the first time under natural conditions, an explicit demonstration that N_2_ fixed by cyanobacteria associated with boreal feather-mosses can follow multiple transfer pathways including through the microbiome (Lindo and Gonzalez, 2010). By doing so, this study demonstrates that the assumption that only decomposition can release newly fixed N_2_ conserved in the moss tissue is not correct and provides compelling evidence that there are rapid transfer pathways through the microbial food webs associated with the feather-moss microbiome of *P. schreberi*. Overall, isotope imaging of ^15^N clearly exposed the complex and contrasting pathways by which newly fixed N_2_ enters and cycles through the boreal forest floor and highlights the need to better understand the ecological role of moss-cyanobacteria associations as part of a more complex boreal bryosphere.

## Data availability statement

The original contributions presented in the study are publicly available. This data can be found here: NCBI, PRJNA376120, SRX2661814-SRX2661825 (ITS1), SRX2661826-SRX2661837 (16S rRNA) and SRX3747711-SRX3747722 (nifH). Representative nifH sequences used for phylogenetic analyses are available from GenBank, accession numbers MH019288- MH019294.

## Author contributions

DM, MA-C, TD, PC, DJ, and JB designed the study. MA-C, TD, and JB performed the research. VS-E, MA-C, PC, and SW prepared moss samples for microscopy, TEM and NanoSIMS analyses and performed 80 nm TEM microscopy analyses. JB performed the NanoSIMS analysis. DC, NC, and MA-C carried out the molecular analysis. DC and NC carried out the bioinformatics analysis. MA-C and JB analyzed the NanoSIMS data. MA-C, JB, and DM wrote the initial draft of the paper. TD, NC, NO, DJ, DC, VS-E, and PC discussed the results and worked on manuscript revisions. All authors contributed to the article and approved the submitted version.
