# Supplementary material for: Revealing the transfer pathways of cyanobacterial-fixed N into the boreal forest through the feather-moss microbiome
Source: Front Plant Sci. 2022 Dec 9;13:1036258. doi: 10.3389/fpls.2022.1036258 (PMC9780503; doi:10.3389/fpls.2022.1036258)
Supplement: Supplementary file 1 [file DataSheet_1.zip › Figure S1.PDF]

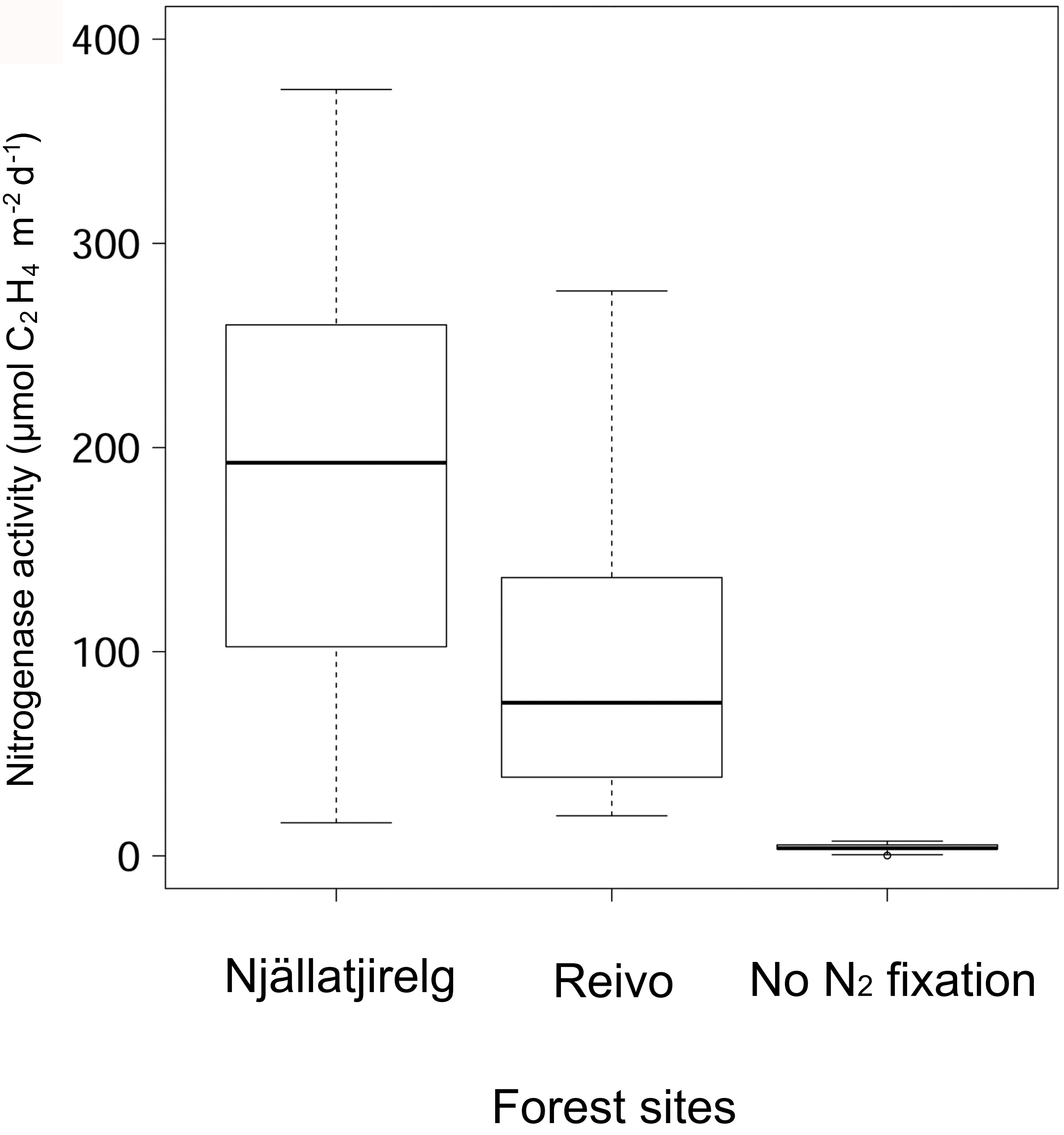


**Fig. S1** Boxplot of nitrogenase activity (as proxy of $N_2$ fixation rates) in *Pleurozium schreberi* ground layer from two different forest sites with different canopy structure and nitrogenase ($N_2$ fixation) activity: Njällatjirelg, an open canopy forest with high forest floor moss $N_2$ fixation and Reivo, a variably dense canopy forest with moderately high $N_2$ fixation in the moss layer; a forest site with no $N_2$ fixation in the moss layer is also included as reference for absence of $N_2$ fixation.
