# Supplementary material for: Revealing the transfer pathways of cyanobacterial-fixed N into the boreal forest through the feather-moss microbiome
Source: Front Plant Sci. 2022 Dec 9;13:1036258. doi: 10.3389/fpls.2022.1036258 (PMC9780503; doi:10.3389/fpls.2022.1036258)
Supplement: Supplementary file 1 [file DataSheet_1.zip › Figure S2.PDF]

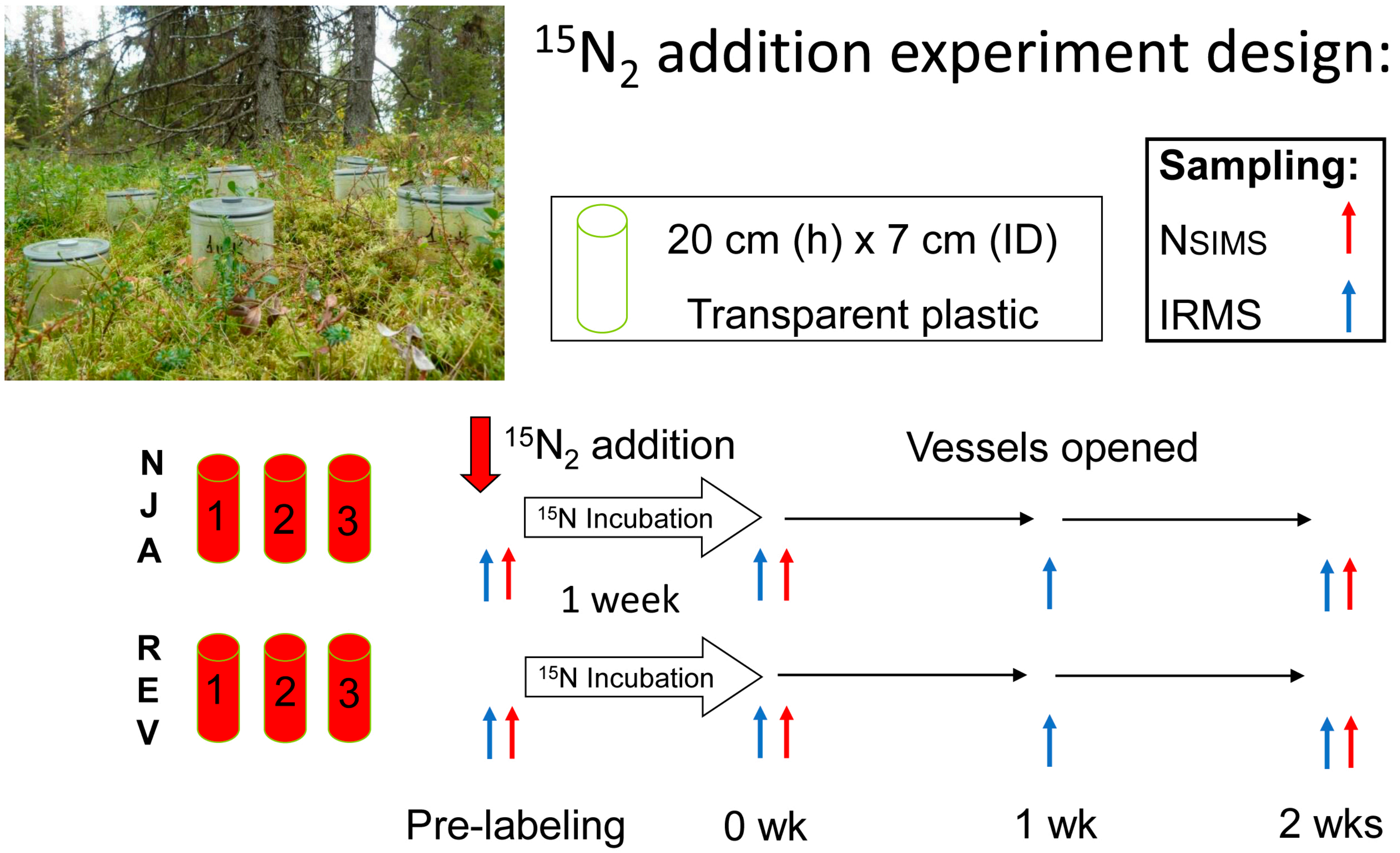

Fig. S2 Diagram of the experimental design. Three moss sample cores, including living and dead parts of the moss shoot, litter layer and humus soil, were collected from two different forest sites at: Njällatjirelg, an open canopy forest with high forest floor moss N_2 fixation (nitrogenase activity) and Reivo, a variably dense canopy forest with moderately high N_2 fixation (nitrogenase activity) in the moss layer (Fig. S1). Moss sample cores were exposed to 100 % headspace $^{15}N_2$ enrichment for one week in individual incubation vessels (20 x 7 cm). All incubation vessels were placed together into holes within the moss layer directly in the field. Moss samples were collected immediately after the incubation ceased (0 wk: one week $^{15}N_2$ exposure) and one and two weeks after the incubation ceased (1wk: 1 week $^{15}N_2$ exposures plus 1 week with open tube; 2wk: 1 week $^{15}N_2$ exposures plus 2 weeks with open tube). Three control samples (6 moss shoots each) for each forest site were collected before the injection of $^{15}N_2$ (pre-labeling) to determine the natural abundance ^{15}N. Sampling time for IRMS (blue) and NanoSIMS (red) analysis are indicated with arrows.
