# Supplementary material for: Revealing the transfer pathways of cyanobacterial-fixed N into the boreal forest through the feather-moss microbiome
Source: Front Plant Sci. 2022 Dec 9;13:1036258. doi: 10.3389/fpls.2022.1036258 (PMC9780503; doi:10.3389/fpls.2022.1036258)
Supplement: Supplementary file 1 [file DataSheet_1.zip › Figure S3.PDF]

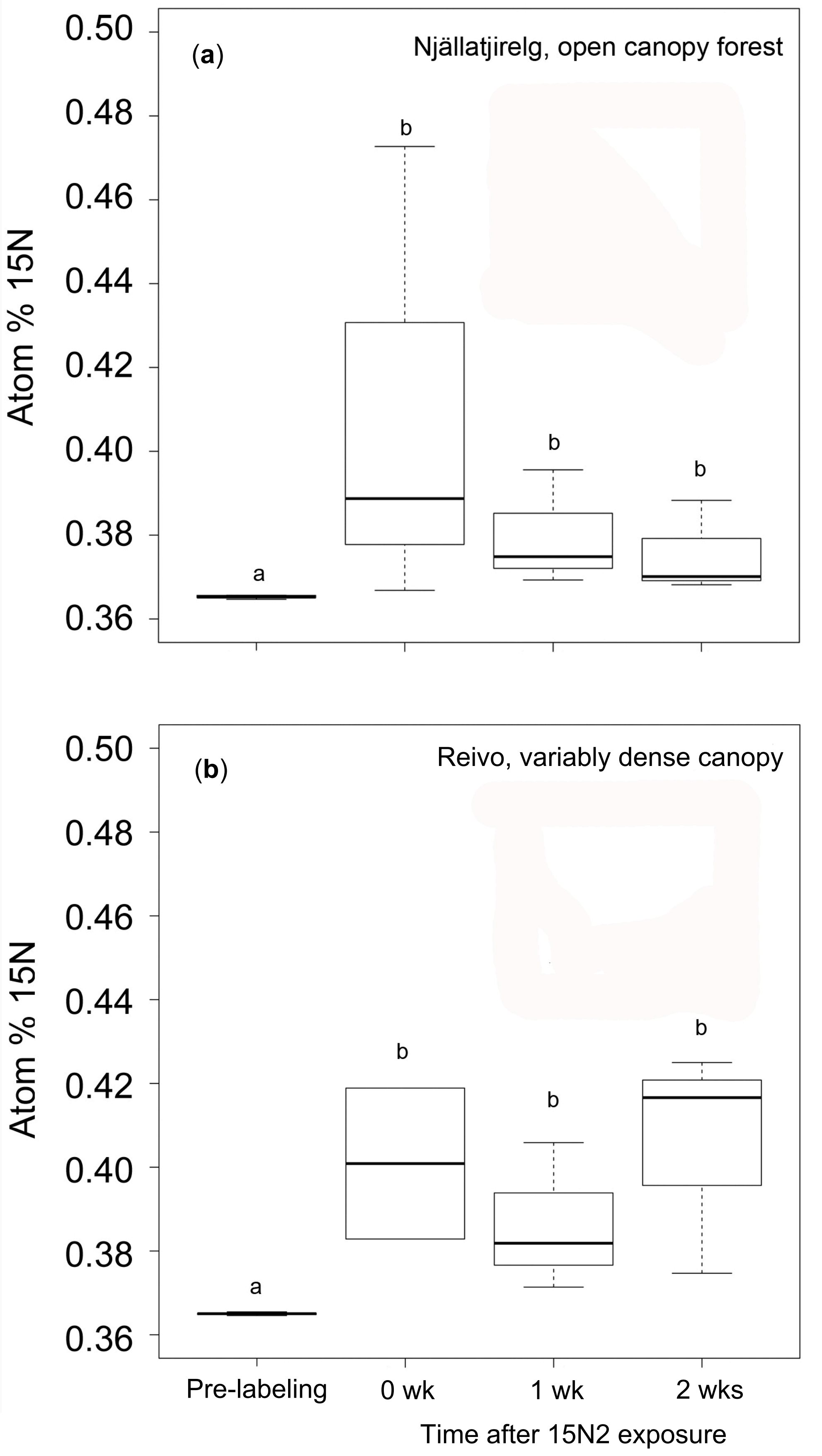

Fig. S3 15N bulk accumulation (15N atom %) in *Pleurozium schreberi* and associated organisms collected from two different forest sites at: (**a**) Njällatjirelg, an open canopy forest with high forest floor moss N2 fixation (nitrogenase activity); (**b**) Reivo, a variably dense canopy forest with moderately high N2 fixation (nitrogenase activity) in the moss layer (Fig. S1). Samples were taken immediately after the $^{15}N_2$ exposure ceased (0 wk: one week $^{15}N_2$ exposure) and one and two weeks after $^{15}N_2$ exposure ceased (1wk: one week $^{15}N_2$ exposures plus one week with open tube; 2wk: one week $^{15}N_2$ exposures plus two weeks with open tube). Pre-labeling indicates bulk 15N natural abundance before $^{15}N_2$ addition. Box and whisker plots encompass 25–75% and 5–95% quantiles of the data, respectively, with the median indicated by a dark horizontal line and outliers shown as dots (n = 3 replicate incubation vessels). Differences between times after $^{15}N_2$ exposure were tested with non-parametric Kruskal-Wallis test followed by pairwise multiple comparison (Dunn´s method). Significant differences between times were found in both forest sites ($P < 0.01$). Pairwise differences between times ($P < 0.05$) are indicated by different letters.
