# Supplementary material for: Revealing the transfer pathways of cyanobacterial-fixed N into the boreal forest through the feather-moss microbiome
Source: Front Plant Sci. 2022 Dec 9;13:1036258. doi: 10.3389/fpls.2022.1036258 (PMC9780503; doi:10.3389/fpls.2022.1036258)
Supplement: Supplementary file 1 [file DataSheet_1.zip › Figure S4.PDF]

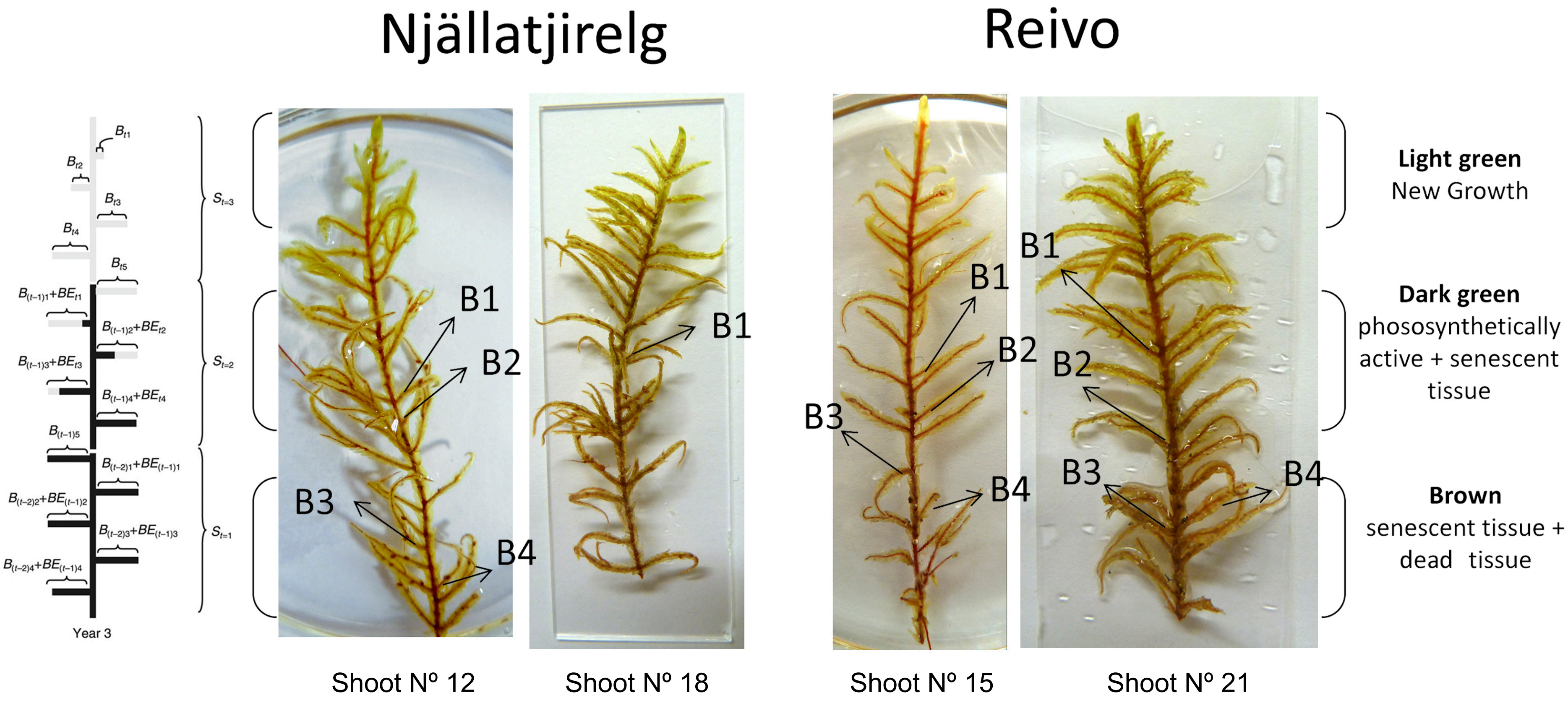

Four selected shoots (from a total of twenty-four) from the highest IRMS 15N enriched samples

Fig. S4 Location of selected branches for NanoSIMS analysis: B1 and B2 for upper dark green segment and B3 and B4 for lower brown segment of 15N enriched moss shoots collected from two different forest sites at: Njällatjirelg, an open canopy forest with high forest floor moss nitrogenase activity (N2 fixation) and Reivo, a variably dense canopy forest with moderately high N2 fixation (Fig. S1). Four moss shoots (from a total of twenty-four) from the highest 15N enriched core samples were selected (shoots Nº 12, 18, 15 and 21). The panel represents a diagram of growth of *P. schreberi* based on Benscoter & Vitt (Journal of Ecology 95: 151-158, 2007).
