# Supplementary material for: Revealing the transfer pathways of cyanobacterial-fixed N into the boreal forest through the feather-moss microbiome
Source: Front Plant Sci. 2022 Dec 9;13:1036258. doi: 10.3389/fpls.2022.1036258 (PMC9780503; doi:10.3389/fpls.2022.1036258)
Supplement: Supplementary file 1 [file DataSheet_1.zip › Figure S5.PDF]

(a) Light microscope image

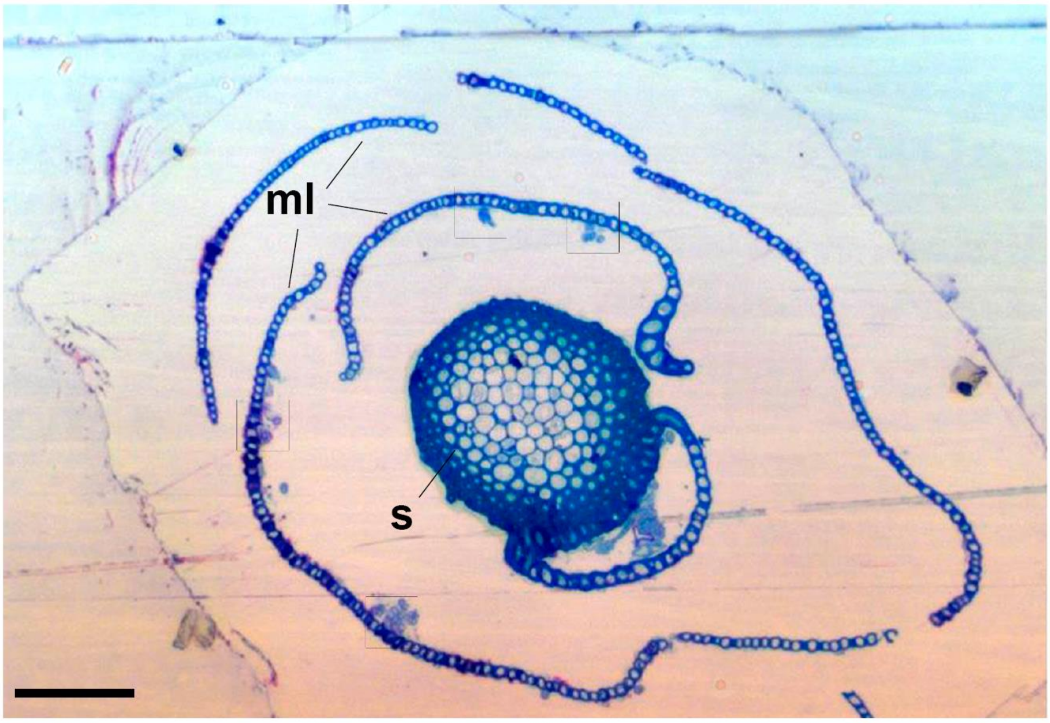

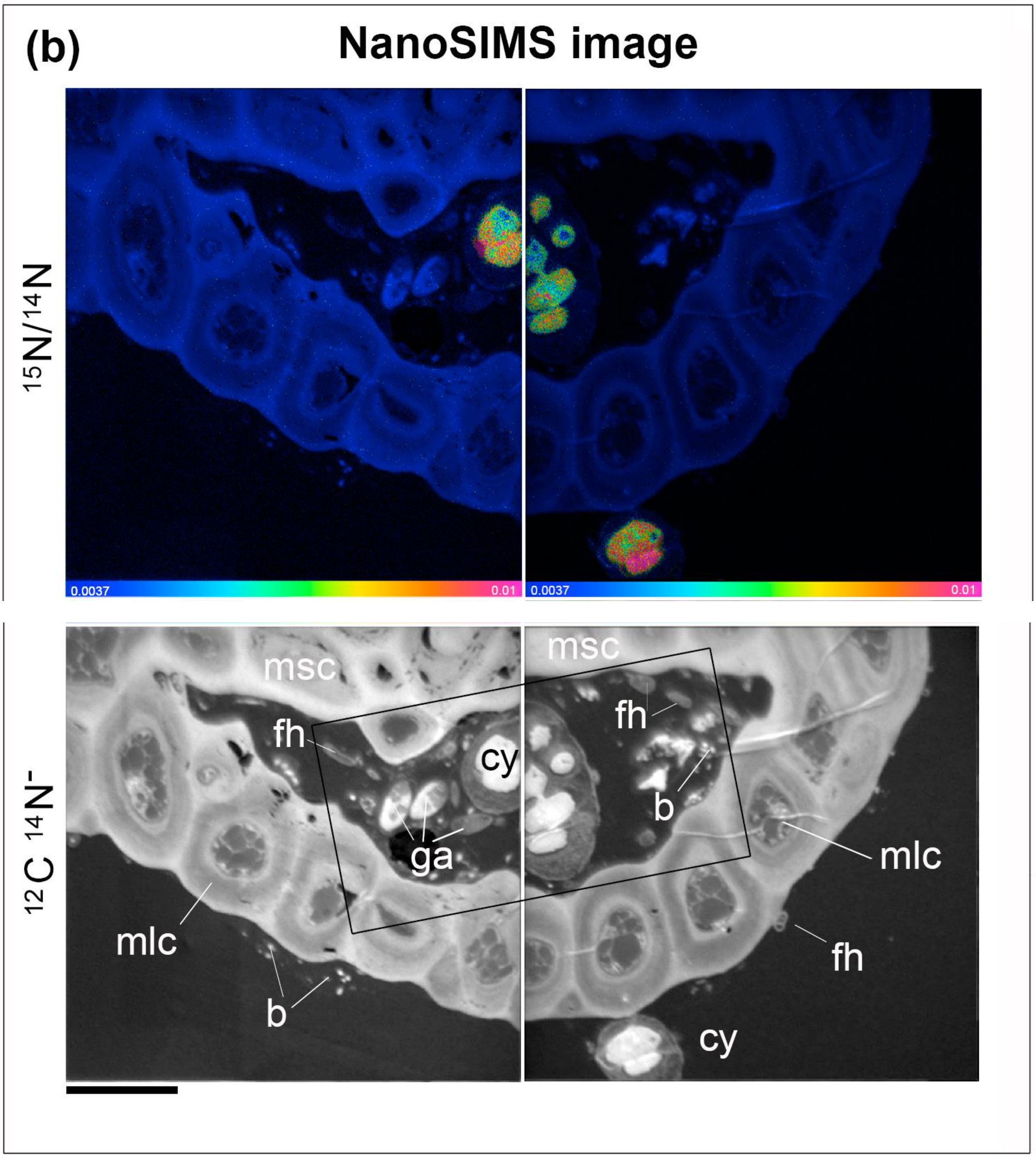
(b)
NanoSIMS image
15N/14N
0.0037
0.01
0.0037
0.01
12C 14N-
msc
msc
fh
fh
cy
b
ga
mlc
mlc
fh
b
cy

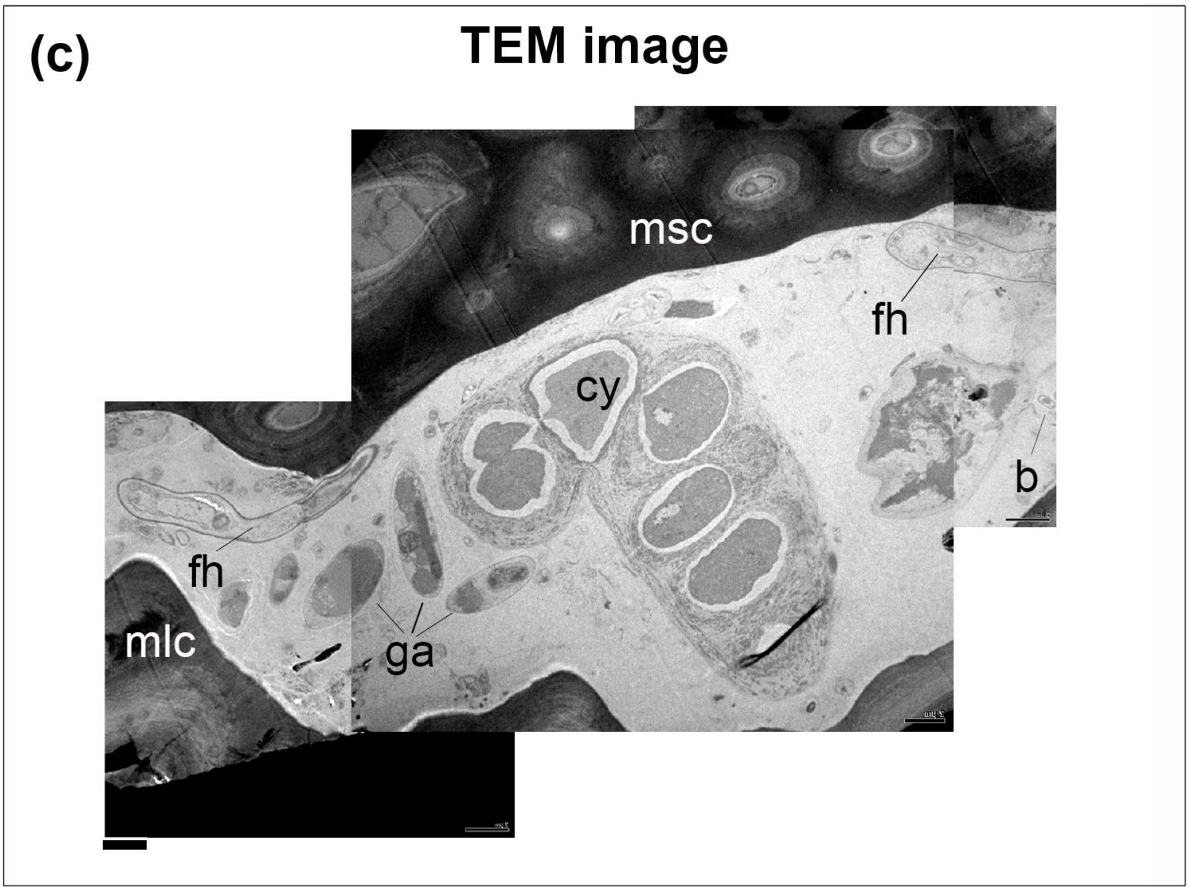

Fig. S5 Representative example of imaging analysis to assess the fate of the newly fixed-N in the boreal bryosphere. Transverse sections through resin-embedded nature branches of *Pleurozium schreberi*. (a) Light microscope images of toluidine blue stained cross-sections showing microbial communities located between moss leaves (ml) and the stem (s), examples of typical regions analysed by NanoSIMS (40 x 40 μm rastered sections) are shown within squares, bar = 100 μm. (b) $^{15}N/^{14}N$ within these regions after one week continuous exposure to $^{15}N_2$ are shown as ^{15}N / ^{14}N Hue-Saturation-Intensity (HSI) images generated from NanoSIMS data, from natural abundance (blue, 0.0037) to enriched (pink, 0.01), with corresponding greyscale $^{12}C^{14}N$ images where cell structures are visible, bar = 10 μm. (c) Correlative transmission electron micrograph (TEM) sections of the same sample showing the cellular ultrastructure of the different components of the bryosphere, bar = 2 μm. Cyanobacteria (cy), bacteria (b), fungal hyphae (fh), green micro-algae (ga), moss stem cell (msc) and moss leaf cell (mlc), bar = 2 μm.
