# Supplementary material for: Revealing the transfer pathways of cyanobacterial-fixed N into the boreal forest through the feather-moss microbiome
Source: Front Plant Sci. 2022 Dec 9;13:1036258. doi: 10.3389/fpls.2022.1036258 (PMC9780503; doi:10.3389/fpls.2022.1036258)
Supplement: Supplementary file 1 [file DataSheet_1.zip › Figure S6.PDF]

Fig. S6 Raw ^{15}N : ^{14}N Hue-Saturation-Intensity (HSI) images of rastered sections generated from NanoSIMS data, from natural abundance (blue, ^{15}N : ^{14}N = 0.0037) to enriched (pink, ^{15}N : ^{14}N = 0.05 to 0.01), with corresponding greyscale $^{12}C^{14}N$ images or correlative greyscale transmission electron micrograph (TEM) images showing the cellular ultrastructure of the different components of the bryosphere (i.e. moss, cyanobacteria, bacteria, fungi and micro-algae cells). Moss samples were collected from two forest sites, Njällatjirelg (an open canopy forest with high rates of forest floor moss N_2 fixation) and Reivo (a variably dense canopy forest with moderately high rates of N_2 fixation). For NanoSIMS analysis, samples were collected before the injection $^{15}N_2$ (control) and immediately after the incubation ceased (0 wk: one week $^{15}N_2$ exposure) and two weeks after the incubation ceased (2wk: 1 week $^{15}N_2$ exposures plus 2 weeks with open tube, Fig. S2). Twenty-four moss shoots (one shoot per sample core and time-point) were fixed for NanoSIMS analysis in parallel to IRMS sampling along the incubation period. We selected the highest bulk (IRMS) ^{15}N enriched samples for high resolution NanoSIMS analysis: 0.47 atom % ^{15}N (0 wk time-point) and 0.39 atom % ^{15}N (2 wk time-point) from Njällatjirelg and 0.42 atom % ^{15}N (0 wk time-point) and 0.42 atom % ^{15}N (2 wk time-point) from Reivo. Six individual branches (location B1 and B2, Fig. S4) from 5 shoots (two from Njällatjirelg and two from Reivo) were analysed across the green portion of moss stems (13 rastered sections and 540 ROIs were analysed in total) and 5 individual branches (location B3 and B4, Fig. S4) from 3 shoots (one from Njällatjirelg and two from Reivo) were analysed across the brown portion of moss stems (10 rastered sections and 352 ROIs were analysed in total). 3 individual branches from 3 shoots (two from Njällatjirelg and one from Reivo) were analysed in unlabelled samples from the green portion of the stem (86 ROIs in 5 rastered sections). The scale bars represents 10 µm.

	Njällatjirelg			Reivo		
	Control<br>0.37 atom %<br>(IRMS 15N enrichment)	0 wk<br>0.47 atom %<br>(highest IRMS 15N enrichment)	2 wks<br>0.39 atom %<br>(highest IRMS 15N enrichment)	Control<br>0.37 atom %<br>(IRMS 15N enrichment)	0 wk<br>0.42 atom %<br>(highest IRMS 15N enrichment)	2 wks<br>0.42 atom %<br>(highest IRMS 15N enrichment)
Dark Green part of the moss shoot: branches B1 and B2 (Fig. S4)	Shoot № 1 – branch B1<br>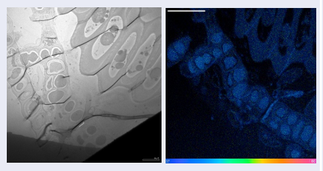 Shoot № 5 – branch B1<br>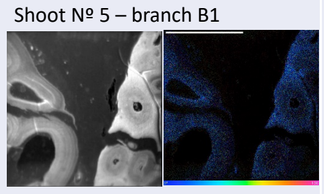 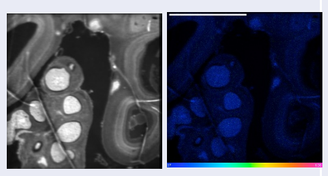 Shoot № 5 – branch B1 	Shoot № 12 – branch B1<br>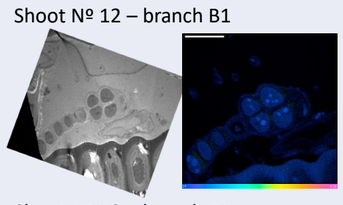 Shoot № 12 – branch B1<br>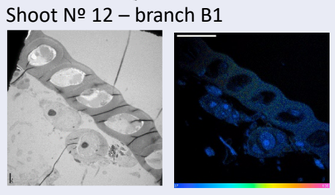 Shoot № 12 – branch B1<br>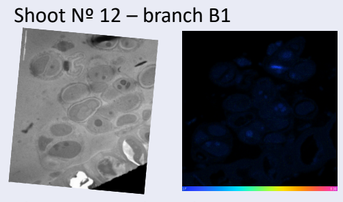 Shoot № 12 – branch B1<br>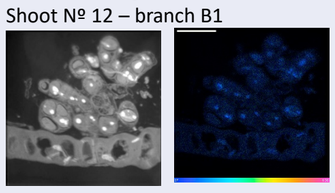 Shoot № 12 – branch B1<br>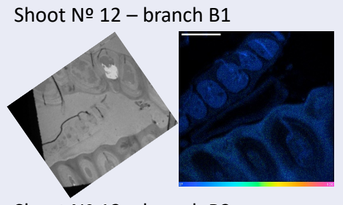 Shoot № 12 – branch B2<br>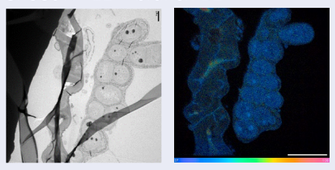 Shoot № 12 – branch B2<br>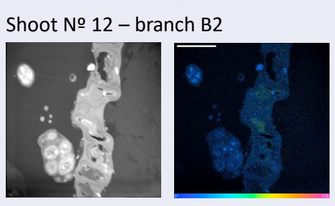 Shoot № 12 – branch B2<br>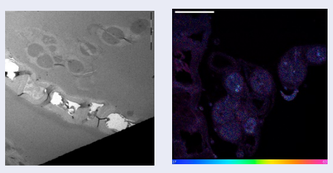	Shoot № 18 – branch B1<br>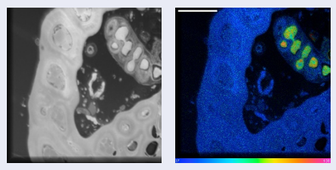 Shoot № 18 – branch B1<br>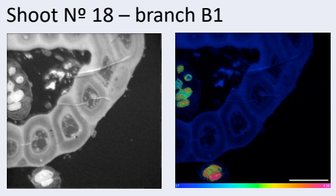	Shoot № 7 – branch B1<br>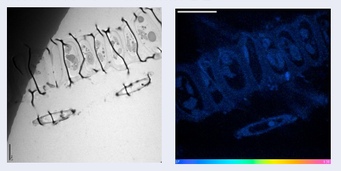 Shoot № 7 – branch B1<br>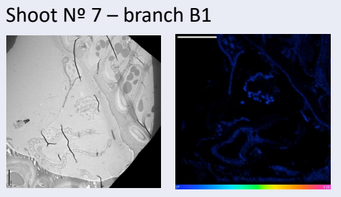	Shoot № 15 – branch B1<br>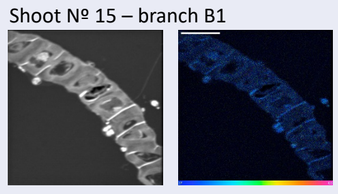 Shoot № 15 – branch B2<br>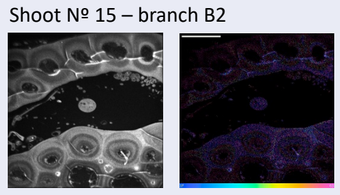	Shoot № 20 – branch B2<br>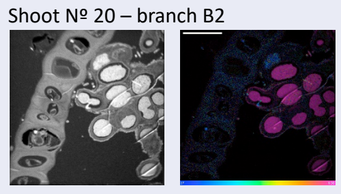
Brown part of the moss shoot: branches B3 and B4 (Fig. S4)		Shoot № 12 – branch B3<br>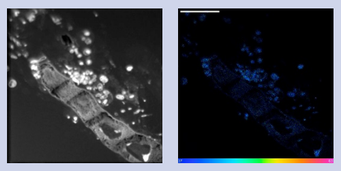 Shoot № 12 – branch B4<br>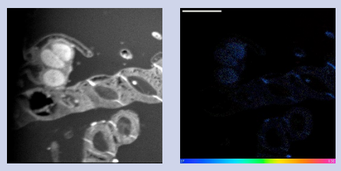 Shoot № 12 – branch B4<br>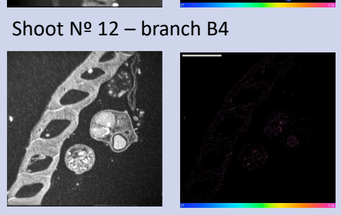			Shoot № 15 – branch B3<br>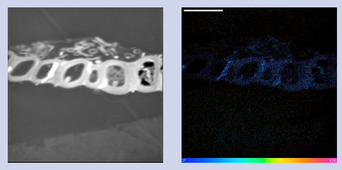	Shoot № 21 – branch B3<br>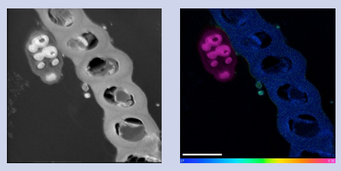 Shoot № 21 – branch B3<br>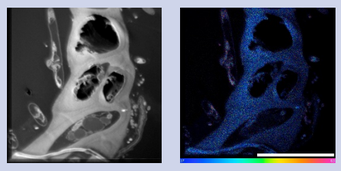 Shoot № 21 – branch B3<br>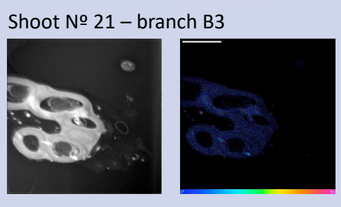 Shoot № 21 – branch B3<br>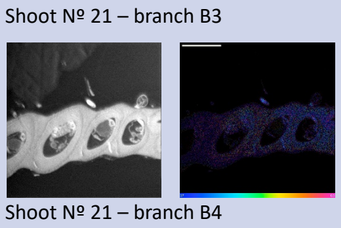 Shoot № 21 – branch B4<br>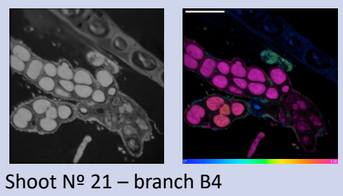 Shoot № 21 – branch B4<br>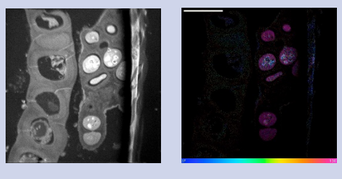
