# Supplementary material for: Revealing the transfer pathways of cyanobacterial-fixed N into the boreal forest through the feather-moss microbiome
Source: Front Plant Sci. 2022 Dec 9;13:1036258. doi: 10.3389/fpls.2022.1036258 (PMC9780503; doi:10.3389/fpls.2022.1036258)
Supplement: Supplementary file 1 [file DataSheet_1.zip › Figure S7.PDF]

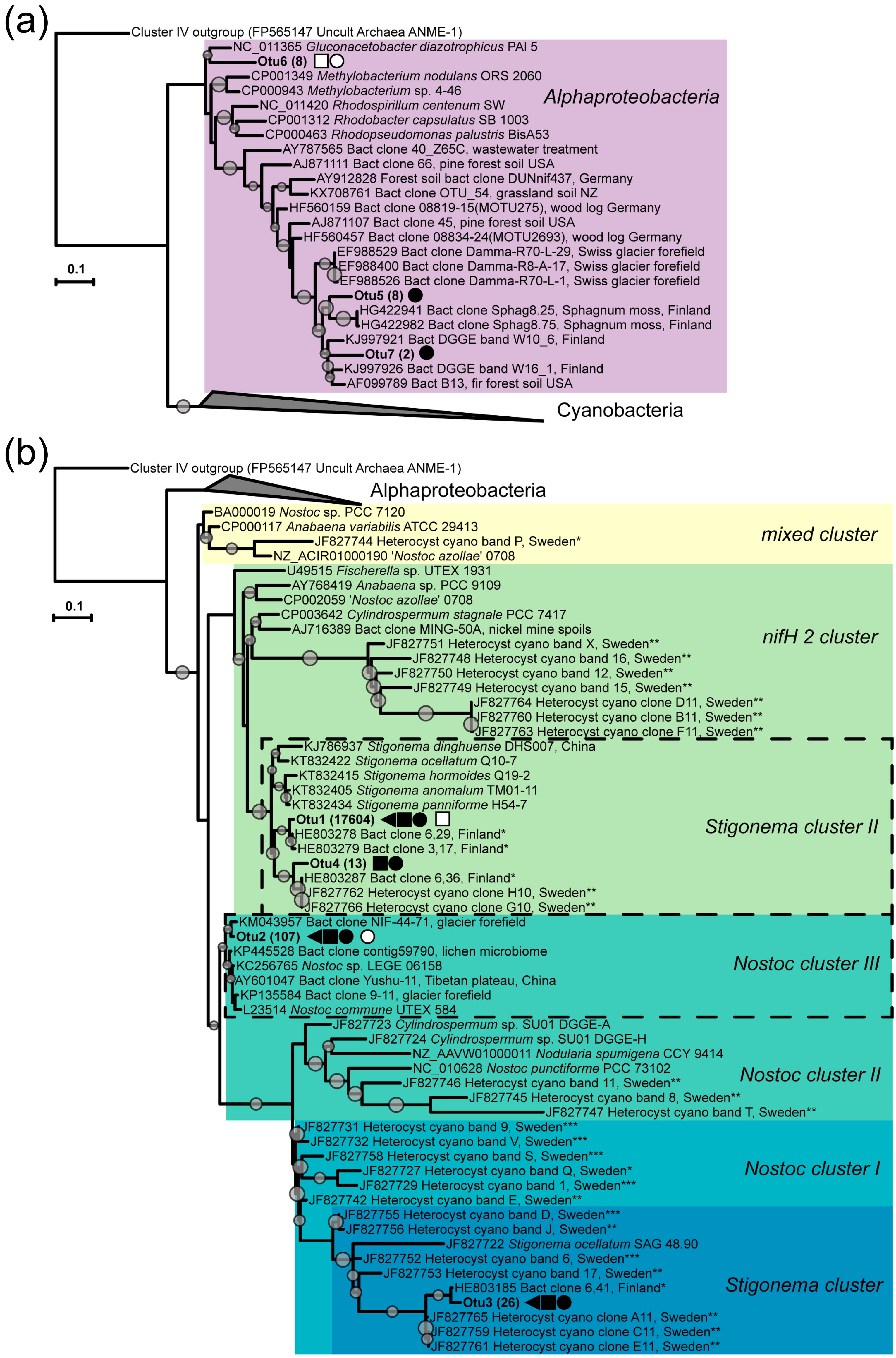

Fig. S7 (a) Phylogenetic tree showing the placement in the class Alphaproteobacteria (*nif*H cluster 1J/1K) of representative sequences from *nif*H OTUs clustered at 90% nucleotide similarity. (b) Phylogenetic tree showing the placement in the phylum Cyanobacteria (*nif*H cluster 1B) of representative sequences from *nif*H OTUs clustered at 90% nucleotide similarity. Numbers in parentheses show the numbers of sequences encompassed by each OTU. Symbols indicate the site and location along the moss stem where sequences were detected: left triangle= new growth tissue from the first 1 cm from the apex, square= mature photosynthetically active segment below the new growth, circle=senescent moss tissue, black= Njällatjirelg (an open canopy forest with high forest floor moss N2 fixation), white= Reivo (a variably dense canopy forest with moderately high N2 fixation). Reference sequences from other boreal feather moss studies are shown with asterisks: *Pleurozium schreberi, **Hylocomium splendens, ***both. Cyanobacteria clusters identified by Ininbergs et al. (2011 are shown in colour, new clusters identified in this study are shown with dashed lines. Trees were computed in ARB using approximately-maximum likelihood with FastTree2, based on 275 nucleotide positions. Nodes with 70-100% support are indicated by grey circles. Short reference sequences (JF8277** 'heterocyst cyano band') did not span the entire alignment and were added to the final tree by quick-add parsimony in ARB.
