# Supplementary material for: Revealing the transfer pathways of cyanobacterial-fixed N into the boreal forest through the feather-moss microbiome
Source: Front Plant Sci. 2022 Dec 9;13:1036258. doi: 10.3389/fpls.2022.1036258 (PMC9780503; doi:10.3389/fpls.2022.1036258)
Supplement: Supplementary file 1 [file DataSheet_1.zip › Figure S8.PDF]

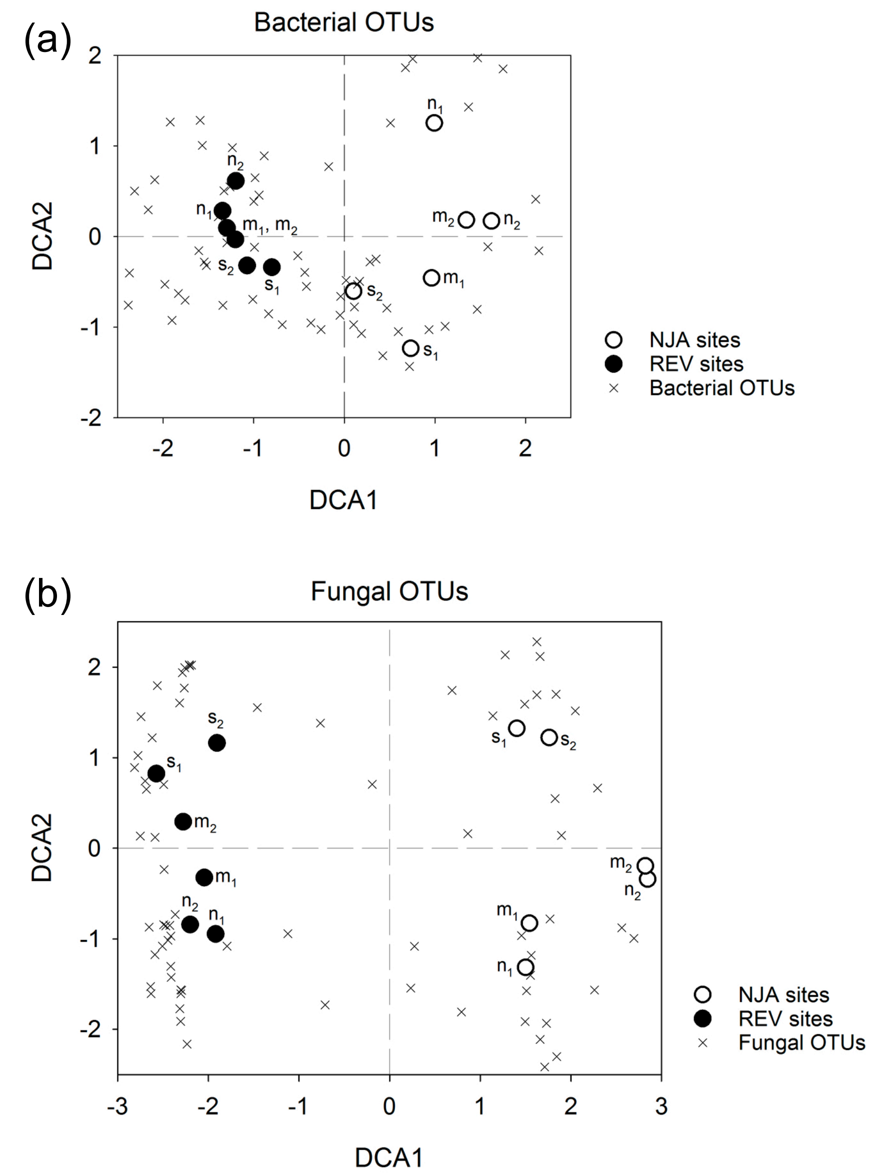

Fig. S8 Detrended correspondence analysis (DCA) of operational taxonomic units (OTUs) for (a) bacteria, 97% similarity for bacterial 16S rRNA, and (b) fungi, 93% similarity for fungal ITS1. Circles show different moss tissue types: ‘light green’ (n) corresponding to new growth tissue from the first 1 cm from the apex, ‘dark green’ (m) to the mature photosynthetically active segment below the new growth and ‘brown/senescent tissue’ (s) corresponding with the senesced segment. Samples were collected at two different sites: Njällatjirelg (NJA), an open canopy forest with high forest floor moss N2 fixation (nitrogenase activity) and Reivo (REV), a variably dense canopy forest with moderately high N2 fixation (nitrogenase activity) in the moss layer. Subscripted numbers refer to the two pooled samples per site (1 and 2 from loca tions 1-6 and 7-12 respectively). Black crosses show the location of the bacterial and fungal OTUs in the biplot.
