# Supplementary material for: Revealing the transfer pathways of cyanobacterial-fixed N into the boreal forest through the feather-moss microbiome
Source: Front Plant Sci. 2022 Dec 9;13:1036258. doi: 10.3389/fpls.2022.1036258 (PMC9780503; doi:10.3389/fpls.2022.1036258)
Supplement: Supplementary file 1 [file DataSheet_1.zip › Figure S9.PDF]

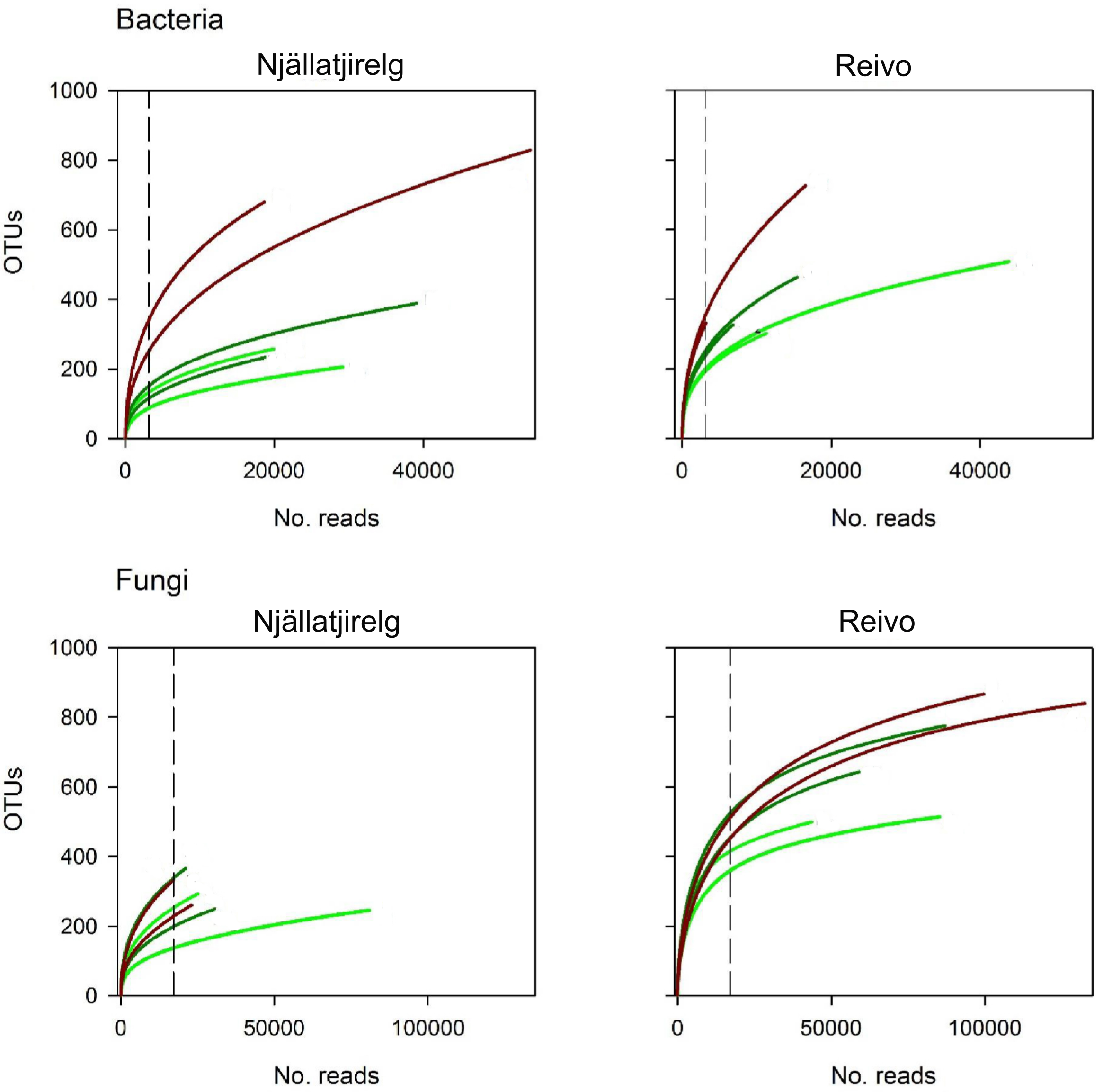


**Fig. S9** Rarefaction curves for the bacterial (a) and fungal (b) OTU data, grouped by site: Njällatjirelg, an open canopy forest with high forest floor moss N2 fixation (nitrogenase activity) and Reivo, a variably dense canopy forest with moderately high N2 fixation (nitrogenase activity) in the moss layer. Colour indicate the location along the moss stem where sequences were detected: light green = new growth tissue from the first 1 cm from the apex, dark green = mature photosynthetically active segment below the new growth, dark red =senescent moss tissue. The standardisation thresholds used are indicated with dashed vertical lines in each case (3190 reads for the bacteria and 17227 reads for the fungi).
