# Supplementary material for: Revealing the transfer pathways of cyanobacterial-fixed N into the boreal forest through the feather-moss microbiome
Source: Front Plant Sci. 2022 Dec 9;13:1036258. doi: 10.3389/fpls.2022.1036258 (PMC9780503; doi:10.3389/fpls.2022.1036258)
Supplement: Supplementary file 1 [file DataSheet_1.zip › Supplementary information.PDF]

## Supplementary Information

# Revealing the transfer pathways of cyanobacterial-fixed N into the boreal forest through the feather-moss microbiome

**María Arróniz-Crespo[1,2], Jeremy Bougoure[3,10], Daniel V. Murphy[3,4], Nick A. Cutler[5,6], Virginia Souza-Egipsy[7], Dominique L. Chaput[8], Davey L. Jones[1,4*], Nicholas Ostle[9], Stephen C. Wade[10], Peta L. Clode[4,11] and Thomas H. DeLuca[12]**

### Methods S1. IRMS measurements and results

Immediately after each collection, samples were oven dried (60ºC for 48 h), ground in a ball mill and sent for $^{15}$N enrichment and total N analysis. All isotopic analyses on moss samples were conducted at the UC Davis Stable Isotope Facility, Department of Plant Sciences, University of California, Davis, USA. $\delta^{15}$N analyses were performed by elemental analyser/continuous flow isotope ratio mass spectrometry (IRMS) using a ANCA-GSL elemental analyser interfaced coupled with a PDZ Europa 20-20 isotope ratio mass spectrometer (Sercon Ltd., Cheshire, UK). Atom% was calculated from $\delta^{15}$N values. Total tissue N was also analysed.

Isotope IRMS results were consistent with measured rates of $N_2$ fixation at each forest sites (Fig. S1). Enrichment of $^{15}$N (> 0.37 atom % $^{15}$N for natural abundance) was found in both forest sites (0.372 – 0.472 for 25–75% quantiles, Fig. S3a,b) and were well within the range of previous reported enrichment for SIMS analyses of diazotrophic systems (Popa et al., 2007; Bay et al., 2013; Woebken et al., 2015). No difference was apparent in bulk-level of $^{15}$N enrichment between incubation time-points (0 wk, 1 wk and 2 wk) for either forest site (Fig. S3). We selected the highest $^{15}$N enriched samples for high resolution NanoSIMS analysis: 0.47 atom % $^{15}$N (0 wk time-point) and 0.39 atom % $^{15}$N (2 wk time-point) from Njällatjirelg forest and 0.42 atom % $^{15}$N (0 wk time-point) and 0.42 atom % $^{15}$N (2 wk time-point) from Reivo forest, (Fig. S3a,b). Three moss shoots (two from Njällatjirelg and one Reivo) from the control samples (collected before the injection $^{15}N_2$) were used as unlabelled controls for NanoSIMS analysis.

### Methods S2. Resin embedding and sectioning

After glutaraldehyde fixing, selected branches from the moss shoots (Fig. S4) were dissected in sections of 2-3 cm: apical, medium and lower areas were identified. All branch sections were post-fixed with 1% (w/v) osmium tetroxide in 0.05 M K-phosphate buffer, pH 7.4 for 5 h at room temperature, dehydrated through an ethanol series and

embedded in Spurr's resin via propylene oxide. After the material had been infiltrated with 100% Spurr's resin several times, it was transferred to a flat silicon mold and covered with fresh resin. Finally, polymerization was performed by placing the samples at 70ºC for 24 h. An Ultracut E ultramicrotome (Reichert-Jung) was used to section embedded material to an initial thickness of 0.35 µm to check for the presence of epiphytic communities of cyanobacteria, fungi and bacteria. This was done by staining sections with toluidine blue followed by examination with a Zeiss Primo Star optical microscope. After identification of appropriate areas, sections for imaging with NanoSIMS and TEM were cut. Thin sections (150 nm) were cut with a diamond knife, placed on carbon coated indexed copper grids and sent to The University of Western Australia for NanoSIMS analysis. Ultrathin sections (80 nm) were then cut in the same sample for TEM imaging, placed on formvar copper mesh grids and stained with lead citrate[1] for 10 min. Both grids were examined at 80 kV with a Leo 910 transmission electron microscope equipped with a Gatan BioScan 792 camera.

## Methods S3. NanoSIMS analysis and image processing

*In situ* isotopic mapping was performed at The University of Western Australia using a NanoSIMS 50 (Cameca, Gennevilliers, France), with a 16 keV $Cs^+$ primary ion beam. Analyses were performed in multi-collection mode with the trolleys positioned to simultaneously detect the negative secondary ions $^{12}C^{14}N$ and $^{12}C^{15}N$. The mass spectrometer was tuned to high mass resolution of c. 10000 (Cameca definition) to separate the $^{12}C^{15}N$ from the $^{13}C^{14}N$ peak on mass 27 using an entrance slit of 30 µm, an aperture slit of 200 µm, and a 10% reduction in the signal at the energy slit. For secondary ion imaging, the primary current was set to c. 20 pA to optimise the secondary ion signal using a 300-µm primary aperture (D1), giving a spot size of c. 100 nm. Images were acquired by rastering the beam over an area 40 × 40 µm square, at a resolution of 256 × 256 pixels, giving a pixel size of 78 nm. TEM micrographs were used to navigate around the sample. Image data collected consisted of the total number of counts for a given secondary ion species recorded on each pixel, with count times kept constant at 60 ms per pixel. All areas were implanted to the same ion dose by the primary beam before each acquisition to remove surface contamination and to enhance the generation of secondary ions. Images were processed using the OpenMIMS data analysis software (National Resource for Imaging Mass Spectrometry http://nrims.harvard.edu) for the freeware package ImageJ (National Institutes of Health, Bethesda, MD, USA). Images were

corrected for detector dead time (44 ns) on individual pixels before any other data processing. Maps representing the $^{15}N$:$^{14}N$ ratios were obtained by dividing the $^{12}C^{15}N$ counts by $^{12}C^{14}N$ counts on each pixel. Numerical $^{15}N$:$^{14}N$ ratio data were extracted directly from the images by selecting regions of interest (ROI: discrete groups of pixels that define a particular feature), and extracting the total number of counts for the given ROI. Ratios were calibrated by taking daily measurements of a *Saccharomyces cerevisiae* standard independently analysed by IRMS ($\delta^{15}N$ = 0.04). The effect of quasi-simultaneous arrivals (QSA;(Slodzian et al., 2001) on the ratios was tested by applying different beta corrections to individual ROIs from several images. Beta values of 1, 0.75 and 0.5 (Hillion et al., 2008) were applied to the data directly using the OpenMIMS software, but were found to have a negligible effect on these data (data not shown). As such, the data presented here were not corrected for QSA.

## Methods S4. Amplicon library preparation

For 16S rRNA and ITS1 based sequencing, triplicate 25 µL reactions consisted of 2.5 µL diluted template DNA per reaction (1.25 ng), 12.5 µL of NEBNext High-Fidelity PCR master mix (New England Biolabs), and 5.0 µL of 1 µM stock of target-specific primers amended with Nextera XT adapters on their 5' end, for a final primer concentration of 0.2 µM. Primers were NGS-grade with PTO modification at the 3' end (Eurofins Genomics). The negative control consisted of 10 mM Tris pH 8.5 buffer, and, for 16S rRNA only, a commercial mock community was amplified as a positive control (ZymoBIOMICS Microbial Community DNA Standard, Zymo Research). Thermocycling conditions were as follows: 95°C for 3 min, 8 cycles of 95°C for 30 s, 50/55°C for 30 s (16S rRNA/ITS1), 72°C for 30 s, and a final elongation at 72°C for 5 min. Triplicate reactions were pooled, cleaned by solid-phase reversible immobilization (SPRI) on carboxylated paramagnetic beads (Thermo Scientific) and eluted in 30 µL TE+Tween buffer (10 mM Tris, 1 mM EDTA, 0.05% Tween 20, pH 8.0). The second PCR was carried out with 15 µL of the cleaned pooled product, 5 µL of each Nextera XT Index primer, and 25 µL of NEBNext High-Fidelity PCR master mix, with the following thermocycling conditions: 95°C for 3 min, 20 cycles of 95°C for 30 s, 55°C for 30 s, 72°C for 30 s, and a final elongation at 72°C for 5 min. PCR products were cleaned as above and eluted in 25 µL TE+Tween buffer. Libraries were quantified with the Qubit dsDNA BR Assay Kit and verified on the Agilent 2200 TapeStation system with High Sensitivity

D1000 ScreenTape (Agilent Technologies). Libraries were diluted to 4 nM and pooled by target. The pools were checked on the TapeStation system and by qPCR. The 16S rRNA amplicon pool needed no further clean-up. The ITS1 pool had residual nonspecific amplicons so was further cleaned with the Qiagen MinElute Gel Extraction Kit according to the manufacturer's instructions, using a 1% agarose gel with SYBR Safe DNA Gel Stain (ThermoFisher Scientific) viewed on a Dark Reader blue LED transilluminator (Clare Chemical Research).

The PolF/PolR primers (Poly et al., 2001) - widely used in *nif*H diversity studies, including those with high-throughput sequencing platforms (Wang et al., 2013; Collavino et al., 2014; Tu et al., 2016) - were shown to have poor coverage *in silic* (Gaby and Buckley, 2012), so three other *nif*H primer pairs were selected for testing based on their broad coverage (Gaby and Buckley, 2012): F2/R6, *nif*H1/*nif*H2 and IGK3/DVV (Table S1). Preliminary tests were carried out in 25 µL reactions with GoTaq G2 Flexi DNA Polymerase (Promega Corporation), using a pure culture of *Anabaena* sp. as positive control, *E. coli* as negative control, and sample REV_s_07-12 as the test environmental sample (the only one with sufficient DNA for multiple test reactions). A range of primer concentrations (0.2-1.2 µM) and annealing temperatures (46-58°C) were tested. F2/R6 failed under all conditions. *nif*H1/*nif*H2 worked well with *Anabaena* sp., but also gave a product with *E. coli* and failed with sample REV_s_07-12. Only the pair IGK3/DVV performed well enough in these preliminary tests to proceed with library preparation, though it required a lower annealing temperature (51°C) than the published value of 58°C and a higher primer concentration (1.0 µM) than the 16S rRNA and ITS1 primer pairs (0.2 µM), likely due to its high degeneracy.

For *nif*H amplicon library preparation, new IGK3/DVV primers were used with fully degenerate bases (N) instead of inosine (I), which is not recognised by the NEBNext high-fidelity polymerase. Library preparation was carried out as described above, but with 1.0 µM primers and an annealing temperature of 51°C in the first PCR. After the second PCR and SPRI clean-up, nifH libraries were diluted to 4 nM and pooled. The TapeStation showed large amounts of nonspecific amplification so the nifH pool was cleaned by gel extraction, as described above.

The three separate 4 nM amplicon pools (16S rRNA, ITS1 and nifH) were then combined and submitted to the Exeter Sequencing Service (University of Exeter, UK) for

paired-end 300 bp sequencing on the Illumina MiSeq platform. PhiX was spiked in at 10% to add diversity.

## Methods S5. Bioinformatic pipeline

Processing of 16S rRNA amplicon data was carried out using mothur 1.38 (Schloss et al., 2009), following the SOP for MiSeq (Kozich et al., 2013) available at www.mothur.org/wiki/MiSeq_SOP, accessed in January 2017. Chimeras were identified using the mothur implementation of UCHIME (Edgar et al., 2011), using the most abundant sequences in each sample as reference. Non-target amplicons (organelles, Eukarya and sequences that could not be classified at the Domain level) were removed, including 239074 sequences identified as chloroplasts. OTUs were clustered at the 97% similarity level, and sequences were classified against the Silva SSU ribosomal database release 123. Sequencing error rate was assessed using the seq.error command in mothur with the sequences obtained from the microbial mock community sample. Processing of ITS1 amplicon data followed similar steps for initial clean-up (assembling forward and reverse reads, removing poorly-assembled contigs and sequences with ambiguous bases or homopolymers longer than 10 bases). ITSx (Bengtsson-Palme et al., 2013) was then used to extract ITS1. Chimeras were identified as above. OTU clustering was carried out as previously described (Cutler et al., 2017), and sequences were classified against the UNITE v6 fungal ITS database (Abarenkov et al., 2010) available in mothur. Taxa that could be resolved to genus level (190 in total, 130,105 reads) were also assigned to putative ecological guilds using the FunGuild tool (Nguyen et al., 2016).

For *nif*H amplicon data, mothur was used to assemble forward and reverse reads, and to remove poorly-assembled contigs and those with ambiguous bases or homopolymers longer than 10 bases. The FrameBot tool in the RDP functional gene pipeline (http://fungene.cme.msu.edu/FunGenePipeline/) was used to translate sequences to amino acids, correct frame shifts caused by sequencing errors, and remove sequences whose translated proteins showed less than 40% similarity to RDP's reference *nif*H protein sequences (Fish et al., 2013; Wang et al., 2013; Cole et al., 2014). Translated protein sequences were aligned with HMMER3 and binned into the main nifH clusters (I-IV) using a classification and regression trees (CART) model (Frank et al., 2016). Cluster IV sequences (*nif*H paralogues not involved in nitrogen fixation) and those that could not be placed in any of the four clusters were removed. In mothur, nucleotide

sequences corresponding to the remaining cluster I-III protein sequences were aligned against a reference alignment built from the curated *nif*H reference database maintained by the Buckley Lab (Gaby and Buckley, 2014) (http://www.css.cornell.edu/faculty/buckley/nifh.htm), which was trimmed to the region spanned by primers IGK3/DVV (positions 358 to 1103 of the alignment, with numbering based on the *nif*H gene of *Azotobacter vinelandii*, GenBank accession M20568). Only sequences that aligned with this region were kept, and primer sequences were removed. Chimeras were identified as described above and removed, along with singletons. Sequence classification was carried out in mothur with a bootstrap cutoff of 60%, using subsets from two reference databases: the Buckley *nif*H database and the *nif*H database maintained by the Zehr Laboratory (Heller et al., 2014) (https://www.jzehrlab.com/nifh), but including only those sequences with taxonomic information. Sequences were clustered into OTUs at the 90% nucleotide similarity level. Single representative sequences were selected from each OTU and imported into ARB (Ludwig et al., 2004), along with their closest relatives in GenBank as determined by BLAST searches. Sequences were aligned to the Zehr *nif*H database. The ARB quick-add parsimony tool was used to find their approximate placement in the *nif*H guide trees included with the Zehr database, and nearest neighbours were selected. Approximately-maximum likelihood trees were computed using the ARB implementation of FastTree2 (Arkin et al., 2010) with the GTR model on 275 nucleotide positions (excluding any columns with gaps). Some of the nearest relatives identified by BLAST searches did not span the full region and were therefore excluded at this stage, though they were subsequently added to the final tree using the ARB quick-add parsimony tool. This included some of the moss-associated cyanobacteria sequences from (Ininbergs et al., 2011). While those obtained by clone libraries spanned the entire region and were used in tree computation, the sequences obtained through DGGE with the PolF/PolR primers were added afterwards. Trees were viewed and annotated with the Interactive Tree of Life (iTOL) tool (Letunic and Bork, 2016).

Individual-based rarefaction curves were produced for each sample, using the vegan package running in R (vegan). As most environmental microbial communities are under-sampled, we also used vegan to extrapolate the OTU data and estimate the 'true' (underlying) richness of the standardised samples, based on the Chao1 metric (Colwell and Coddington, 1994). For bacterial communities, rarefaction analysis indicated that

moss segments had not been sampled to the point of saturation (Fig. S9). Observed richness in the standardized samples was, on average, about 60% of estimated community richness (Table S6). The rarefaction curves did not cross after the standardisation thresholds (indicated by the vertical dotted lines in Fig. S9). For fungal communities, richness in the standardized samples underestimated underlying community diversity (Table S6). Chao 1 estimates indicated that observed richness in the standardized samples was, on average, about 70% of extrapolated community richness. Except for the fungal samples from Reivo, the rarefaction curves did not cross after the standardisation thresholds (Fig. S9).

## REFERENCES


Abarenkov K, Nilsson RH, Larsson K-H, Alexander IJ, Eberhardt U, Erland S, Hoiland K, Kjoller R, Larsson E, Pennanen T, et al., 2010. The UNITE database for molecular identification of fungi - recent updates and future perspectives. *New Phytologist* 186 281-285.

Arkin AP, Dehal PS, Price MN 2010. FastTree 2 - Approximately Maximum-Likelihood Trees for Large Alignments, Figshare.

Bay G, Nahar N, Oubre M, Whitehouse MJ, Wardle DA, Zackrisson O et al., 2013. Boreal feather mosses secrete chemical signals to gain nitrogen. *New Phytologist* 200, 54-60.

Bengtsson-Palme J, Ryberg M, Hartmann M, Branco S, Wang Z, Godhe A, De Wit P, Sanchez-Garcia M, Ebersberger I, de Sousa F, et al. 2013. Improved software detection and extraction of ITS1 and ITS2 from ribosomal ITS sequences of fungi and other eukaryotes for analysis of environmental sequencing data. *Methods in Ecology and Evolution* 4, 914-919.

Cole JR, Wang Q, Fish JA, Chai BL, McGarrell DM, Sun YN, Brown CT, Porras-Alfaro A, Kuske CR, Tiedje JM. 2014. Ribosomal Database Project: data and tools for high throughput rRNA analysis. *Nucleic Acids Research* 42, D633-D642.

Colwell RK, Coddington JA. 1994. Estimating terrestrial biodiversity through extrapolation. *Philosophical Transactions of the Royal Society of London Series B-Biological Sciences* 345, 101-118.

Collavino MM, Tripp HJ, Frank IE, Vidoz ML, Calderoli PA, Donato M, Zehr JP, Aguilar OM. 2014. nifH pyrosequencing reveals the potential for location-

specific soil chemistry to influence $N_2$-fixing community dynamics. *Environmental Microbiology* 16, 3211-3223.

Cutler NA, Arroniz-Crespo M, Street LE, Jones DL, Chaput DL, DeLuca TH. 2017. Long-Term recovery of microbial communities in the boreal bryosphere following fire disturbance. *Microbial Ecology* 73, 75-90.

Edgar RC, Haas BJ, Clemente JC, Quince C, Knight R. 2011. UCHIME improves sensitivity and speed of chimera detection. *Bioinformatics* 27, 2194-2200.

Fish JA, Chai B, Wang Q, Sun Y, Brown CT, Tiedje JM, Cole JR. 2013. FunGene, the functional gene pipeline and repository. *Frontiers in Microbiology* 4, 291.

Frank IE, Turk-Kubo KA, Zehr JP. 2016. Rapid annotation of nifH gene sequences using classification and regression trees facilitates environmental functional gene analysis. *Environmental Microbiology Reports* 8, 905-916.

Gaby JC, Buckley DH. 2012. A comprehensive evaluation of PCR primers to amplify the nifH gene of nitrogenase. *Plos One* 7, 12.

Gaby JC, Buckley DH. 2014. A comprehensive aligned nifH gene database, a multipurpose tool for studies of nitrogen- fixing bacteria. *Database-the Journal of Biological Databases and Curation* 2014, bau001.

Heller P, Tripp HJ, Turk-Kubo K, Zehr JP. 2014. ARBitrator, a software pipeline for on-demand retrieval of auto-curated nifH sequences from GenBank. *Bioinformatics* 30, 2883-2890.

Hillion F, Kilburn MR, Hoppe P, Messenger S, Weber PK. 2008. The effect of QSA on S, C, O and Si isotopic ratio measurements. *Geochimica et Cosmochimica Acta* 72, A377-A377.

Ininbergs K, Bay G, Rasmussen U, Wardle DA, Nilsson MC. 2011. Composition and diversity of nifH genes of nitrogen-fixing cyanobacteria associated with boreal forest feather mosses. *New Phytologist* 192, 507-517.

Kozich JJ, Westcott SL, Baxter NT, Highlander SK, Schloss PD. 2013. Development of a dual-index sequencing strategy and curation pipeline for analyzing amplicon sequence data on the MiSeq Illumina Sequencing Platform. *Applied and Environmental Microbiology* 79, 5112-5120.

Letunic I, Bork P. 2016. Interactive tree of life (iTOL) v3: An online tool for the display and annotation of phylogenetic and other trees. *Nucleic Acids Research* 44, W242-W245.

Ludwig W, Strunk O, Westram R, Richter L, Meier H, Yadhukumar, Buchner A, Lai T, Steppi S, Jobb G, et al., 2004. ARB: A software environment for sequence data. *Nucleic Acids Research* 32, 1363-1371.

Nguyen NH, Song Z, Bates ST, Branco S, Tedersoo L, Menke J, Schilling JS, Kennedy PG. 2016. FUNGuild: An open annotation tool for parsing fungal community datasets by ecological guild. *Fungal Ecology* 20, 241-248.

Poly F, Monrozier LJ, Bally R. 2001. Improvement in the RFLP procedure for studying the diversity of nifH genes in communities of nitrogen fixers in soil. *Research in Microbiology* 152, 95-103.

Popa R, Weber PK, Pett-Ridge J, Finzi JA, Fallon SJ, Hutcheon ID et al. 2007. Carbon and nitrogen fixation and metabolite exchange in and between individual cells of Anabaena oscillarioides. *ISME Jouranl* 1, 354-360.

Schloss PD, Westcott SL, Ryabin T, Hall JR, Hartmann M, Hollister EB, Lesniewski RA, Oakley BB, Parks DH, Robinson CJ, et al., 2009. Introducing mothur, open-source, platform-independent, community-supported software for describing and comparing microbial communities. *Applied and Environmental Microbiology* 75, 7537-7541.

Slodzian G, Chaintreau M, Dennebouy R, Rousse A. 2001. Precise in situ measurements of isotopic abundances with pulse counting of sputtered ions. *European Physical Journal-Applied Physics* 14, 199-231.

Tu Q, Zhou X, He Z, Xue K, Wu L, Reich P, Hobbie S, Zhou J. 2016. The diversity and co-occurrence patterns of $N_2$-fixing communities in a $CO_2$-enriched grassland ecosystem. *Microbial Ecology* 71, 604-615.

vegan, Community Ecology Package v. 2.2-1. 2015.

Wang Q, Quensen JF, III, Fish JA, Lee TK, Sun Y, Tiedje JM, Cole JR. 2013. Ecological patterns of nifh genes in four terrestrial climatic zones explored with targeted metagenomics using FrameBot, a new informatics tool. *Mbio* 4, e00592-13.

Woebken D, Burow LC, Behnam F, Mayali X, Schintlmeister A, Fleming ED et al. 2015. Revisiting $N_2$ fixation in Guerrero Negro intertidal microbial mats with a functional single-cell approach. *ISME Journal* 9, 485-496.

## SUPPLEMENTARY FIGURES

**Figure S1 |** Studied sites. Boxplot of nitrogenase activity (as proxy of $N_2$ fixation rates) in *Pleurozium schreberi* ground layer from two different forest sites selected in the present study.

**Figure S2 |** Incubation experiment. Diagram of the experimental design.

**Figure S3 |** IRMS results. Bulk-levels of $^{15}N$ enrichment of bryosphere samples from each forest site (*i.e.* Njällatjirelg and Reivo) analysed along the incubation period.

**Figure S4 |** Location of selected branches for NanoSIMS analysis.

**Figure S5 |** Representative example of imaging analysis to assess the fate of the newly fixed-N in the boreal bryosphere.

**Figure S6 |** $^{15}N$:$^{14}N$ Hue-Saturation-Intensity (HSI) images and $^{12}C^{14}N$ images of all rastered sections generated from NanoSIMS data.

**Figure S7 |** Phylogenetic tree from *nif*H sequences.

**Figure S8 |** Detrended correspondence analysis (DCA) of operational taxonomic units (OTUs) for (a) bacteria, 97% similarity for bacterial 16S rRNA, and (b) fungi, 93% similarity for fungal ITS1.

**Figure S9 |** Rarefaction curves for the bacterial and fungal OTU data.

**Table S1** PCR primers used in this study.

**Table S2** Bacterial community composition of the most abundant taxa with depth across the different moss tissue type.

**Table S3** Fungal community composition of the most abundant taxa with depth across the different moss tissue type.

**Table S4** Abundance and putative trophic mode of fungal taxa.

**Table S5** Mycorrhizal fungi abundance with depth across the different moss tissue type.

**Table S6** Chao1 estimates of underlying community richness.
