# Supplementary material for: Revealing the transfer pathways of cyanobacterial-fixed N into the boreal forest through the feather-moss microbiome
Source: Front Plant Sci. 2022 Dec 9;13:1036258. doi: 10.3389/fpls.2022.1036258 (PMC9780503; doi:10.3389/fpls.2022.1036258)
Supplement: Supplementary file 1 [file DataSheet_1.zip › Table S1.PDF]

**Table S1** PCR primers used in this study

| Primer | Target | Sequence** (5' -> 3') | Reference |
|---|---|---|---|
| 515fB | 16S rRNA | GTGYCAGCMGCCGCGGTAA | Parada et al. 2016 |
| 806rB | 16S rRNA | GGACTACNVGGGTWTCTAAT | Apprill et al. 2015 |
| ITS1Fngs | Fungal ITS1 | GGTCATTTAGAGGAAGTAA | Tedersoo et al. 2015 |
| ITS2 | ITS1 | GCTGCGTTCTTCATCGATGC | White et al. 1990 |
| IGK3* | nifH | GCIWTHTAYGGIAARGGIGGIATHGGIAA | Ando et al. 2005 |
| DVV* | nifH | ATIGCRAAICCICCRCAIACIACRTC | Ando et al. 2005 |
| nifH1 | nifH | ADNGCCATCATYTCNCC | Zehr and McReynolds 1989 |
| nifH2 | nifH | TGYGAYCCNAARGCNGA | Zehr and McReynolds 1989 |
| F2 | nifH | TGYGAYCCIAAIGCIGA | Marusina et al. 2001 |
| R6 | nifH | GCCATCATYTCICCIGA | Marusina et al. 2001 |

*Published primer sequence contains inosine (I), but due to incompatibility with NEBNext polymerase, fully degenerate bases (N) were used instead for final amplicon library preparation.

** Only the target-specific primer sequence is shown. Primers included Nextera XT adapters on their 5' end: TCGTCGGCAGCGTCAGATGTGTATAAGAGACAG for forward primers and GTCTCGTGGGCTCGGAGATGTGTATAAGAGACAG for reverse primers.

References:


- Ando, S. et al. Detection of nifH sequences in sugarcane (Saccharum officinarum L.) and pineapple (Ananas comosus [L.] Merr.). Soil Sci. Plant Nutr. 51, 303–308 (2005).
- Apprill, A., Mcnally, S., Parsons, R. & Weber, L. Minor revision to V4 region SSU rRNA 806R gene primer greatly increases detection of SAR11 bacterioplankton. Aquat. Microb. Ecol. 75, 129–137 (2015).
- Marusina, A. et al. A system of oligonucleotide primers for the amplification of nifH genes of different taxonomic groups of prokaryotes. Mikrobiologiya 70, 86–91 (2001).
- Parada, A. E., Needham, D. M. & Fuhrman, J. A. Every base matters: Assessing small subunit rRNA primers for marine microbiomes with mock communities, time series and global field samples. Environ. Microbiol. 18, 1403–1414 (2016).
- Tedersoo, L. et al. Shotgun metagenomes and multiple primer pair-barcode combinations of amplicons reveal biases in metabarcoding analyses of fungi. MycoKeys 10, 1–43 (2015).
- White, T. J., Bruns, T., Lee, S. & Taylor, J. W. in PCR Protocols: A Guide to Methods and Applications (eds. Innis, M. A., Gelfand, D. H., Sninsky, J. J. & White, T. J.) 315–322 (Academic Press, Inc., 1990).
- Zehr, J. P. & McReynolds, L. A. Use of degenerate oligonucelotides for amplification of the nifH gene from the marine cyanobacterium Trichodemium thiebautii. Appl. Environ. Microbiol. 55, 2522–2526 (1989).
