# Supplementary material for: Revealing the transfer pathways of cyanobacterial-fixed N into the boreal forest through the feather-moss microbiome
Source: Front Plant Sci. 2022 Dec 9;13:1036258. doi: 10.3389/fpls.2022.1036258 (PMC9780503; doi:10.3389/fpls.2022.1036258)
Supplement: Supplementary file 1 [file DataSheet_1.zip › Table S2.PDF]

	Njällatjirelg						Reivo					
	n1	n2	m1	m2	s1	s2	n1	n2	m1	m2	s1	s2
**Acidobacteria**												
Acidobacteriales												
Acidobacteriaceae												
Granulicella sp.	2,57	1,35	4,73	2,13	5,17	6,55	14,20	11,57	11,66	12,66	10,88	12,26
Other Acidobacteriaceae	2,92	0,75	4,08	2,32	3,57	6,39	3,23	4,58	6,49	3,92	5,58	4,92
other Acidobacteria	0,06	0,06	0,22	0,09	0,66	0,75	0,09	0,50	0,60	0,97	1,41	1,29
**Actinobacteria**												
Corynebacteriales												
Mycobacteriaceae												
Mycobacterium sp.	0,00	0,00	0,09	0,06	0,91	6,71	0,22	0,06	0,38	0,25	1,76	0,60
Micrococcales												
Microbacteriaceae	7,68	0,66	3,48	0,75	0,85	1,97	1,22	0,69	0,66	0,69	0,53	0,53
other Micrococcales	0,13	0,25	0,06	0,09	0,00	0,00	0,00	0,00	0,00	0,00	0,00	0,00
Solirubrobacterales												
Conexibacteraceae												
Conexibacter sp.	0,19	0,19	0,47	0,50	2,01	5,02	0,56	0,25	1,60	0,69	2,10	1,29
other Solirubrobacterales	0,00	0,03	0,06	0,00	0,22	0,44	0,00	0,00	0,22	0,19	0,38	0,41
other Actinobacteria	2,19	0,22	1,00	0,69	3,64	8,93	5,92	4,92	9,03	8,53	8,43	6,74
u/c Actinobacteria	0,00	0,00	0,00	0,00	0,78	3,61	0,00	0,00	0,13	0,03	0,09	0,22
**Bacteriodetes**												
Sphingobacteriales												
Sphingobacteriaceae												
Mucilaginibacter sp.	0,88	0,06	0,34	0,03	1,13	1,54	1,38	1,57	2,60	1,63	3,26	3,70
other Sphingobacteriaceae	3,45	0,13	0,09	0,03	0,19	0,19	0,60	1,85	0,78	0,16	0,34	0,41
other Bacteriodetes	0,03	0,00	0,03	0,00	0,31	1,76	0,47	0,94	0,28	0,44	0,85	1,19
u/c Bateriodetes	0,00	0,00	0,00	0,00	0,09	0,06	0,00	0,03	0,00	0,00	0,09	0,06
**Cyanobacteria**												
Subsection IV	0,09	0,00	26,30	2,13	28,03	1,00	0,00	0,00	0,00	0,00	0,38	0,00
other Cyanobacteria	0,00	0,00	0,00	0,00	0,03	0,22	0,03	0,03	0,06	0,06	0,19	0,06
u/c Cyanobacteria	0,00	0,00	0,06	0,00	0,03	0,03	0,00	0,00	0,03	0,00	0,06	0,00
**Planctomycetes**												
Planctomycetales												
Planctomycetaceae												
Singulisphaera sp.	0,28	0,16	0,75	0,63	0,94	1,88	0,31	1,00	2,01	1,03	2,66	1,79
Other Planctomycetaceae	0,03	0,06	0,06	0,03	0,22	1,07	0,09	0,09	0,38	0,44	0,66	0,97
WD21010 soil group	0,13	0,06	0,38	0,09	1,22	1,54	0,41	0,63	1,47	0,91	2,26	2,35
**Proteobacteria**												
Alphaproteobacteria												
Caulobacterales												
Caulobacteraceae												
Brevundimonas sp.	0,09	0,06	0,22	0,13	0,09	0,00	0,78	0,66	1,16	1,29	1,16	1,32
Phenylobacterium sp.	0,06	0,03	0,03	0,16	0,31	0,53	0,31	0,28	0,69	0,75	1,63	1,13
Other Caulobacteraceae	0,06	0,03	0,25	0,03	0,25	0,19	0,44	0,03	1,54	0,53	0,53	0,66
Rhizobiales	2,85	0,97	2,13	1,35	3,61	6,74	10,72	14,86	10,16	12,88	6,68	8,56
Rhodospirillales												
Acetobacteraceae												
Acidiphilium sp.	2,16	0,25	1,22	0,22	0,00	0,00	6,46	6,24	2,45	7,30	0,66	1,63
Acidisphaera sp.	0,97	0,60	1,38	0,85	2,07	2,26	0,41	0,22	1,07	0,82	2,38	1,57
Other Acetobacteraceae	5,17	9,28	8,37	11,57	5,96	5,61	16,71	13,01	17,77	17,84	15,86	16,99
Other Rhodospirillales	0,22	0,03	0,16	0,13	0,19	0,66	0,63	0,78	0,50	0,56	0,85	0,66
Sphingomonadales	0,75	0,09	0,31	0,00	0,19	0,16	2,57	1,79	1,44	0,97	1,13	1,03
other Alphaproteobacteria	0,00	0,03	0,00	0,00	0,00	0,00	0,13	0,13	0,13	0,06	0,06	0,03
u/c Alphaproteobacteria	5,20	0,16	1,57	0,50	0,38	0,22	1,76	0,85	1,54	0,38	0,78	0,72
Betaproteobacteria												
Burkholderiales												
Burkholderiaceae												
Burkholderia sp.	6,90	32,82	13,54	31,66	21,29	11,38	0,53	0,34	0,50	0,28	4,51	0,53
Comamonadaceae	0,97	0,34	0,47	0,44	0,34	0,31	5,08	5,61	2,19	3,01	2,23	2,23
Other Burkholderiales	8,59	31,66	7,12	13,42	0,75	3,48	0,06	5,99	0,06	0,09	0,28	0,09
u/c Betaproteobacteria	0,85	0,94	0,31	0,34	0,41	0,56	9,50	3,67	5,05	3,67	1,54	2,63
Gammaproteobacteria												
Pseudomonadales												
Pseudomonadaceae												
Pseudomonas sp.	39,62	16,33	14,14	25,61	0,44	0,03	0,00	1,72	0,00	0,00	0,03	0,00
Xanthomonadales												
Xanthomonadaceae												
Rhodanobacter sp.	1,94	0,00	1,29	0,22	0,60	0,66	0,16	1,91	0,19	0,38	0,31	0,38
Other Xanthomonadales	0,00	0,00	0,06	0,00	0,09	0,38	0,09	0,03	0,13	0,03	0,31	0,60
other Gammaproteobacteria	0,03	1,19	0,00	0,06	0,09	0,19	0,03	0,13	0,00	0,09	0,06	0,13
u/c Gammaproteobacteria	0,72	0,38	1,29	1,00	1,22	3,57	7,30	6,02	6,74	7,84	6,30	7,59
u/c Proteobacteria	0,03	0,03	0,00	0,03	0,06	0,22	0,00	0,25	0,06	0,09	0,03	0,03
Other Proteobacteria	0,09	0,00	0,06	0,25	0,41	0,75	0,25	0,16	0,50	0,47	1,00	0,91
**Verrucomicrobia**												
Chthoniobacterales	0,82	0,03	0,63	0,13	1,25	0,60	4,80	2,76	3,39	2,41	2,41	1,69
Other Verrucomicrobia	0,60	0,06	0,63	0,00	0,50	1,22	1,03	1,85	1,07	1,57	1,13	1,19
**u/c WD272**	0,53	0,60	2,32	2,23	5,17	4,36	0,91	1,38	2,26	3,23	4,08	5,89
**u/c Bacteria**	0,06	0,09	0,19	0,03	3,95	5,08	0,50	0,50	0,78	0,69	1,79	2,54
**Other bacterial phyla**	0,06	0,00	0,09	0,06	0,38	1,19	0,09	0,13	0,28	0,16	0,34	0,50

Table S2 Bacterial community composition of the most abundant taxa with depth across the different moss tissue type: 'light green' (n) corresponding to new growth tissue from the first 1 cm from the apex, 'dark green' (m) to the mature photosynthetically active segment below the new growth and 'brown/senescent tissue' (s) corresponding to the senesced segment. Samples were collected at two different sites: Njällatjirelg, an open canopy forest with high forest floor moss nitrogenase activity (N2 fixation); Reivo, a variably dense canopy forest with moderately high N2 fixation in the moss layer. Subscripted number refers the two pooled samples per site (1 and 2 from locations 1-6 and 7-12 respectively).
