# Supplementary material for: Revealing the transfer pathways of cyanobacterial-fixed N into the boreal forest through the feather-moss microbiome
Source: Front Plant Sci. 2022 Dec 9;13:1036258. doi: 10.3389/fpls.2022.1036258 (PMC9780503; doi:10.3389/fpls.2022.1036258)
Supplement: Supplementary file 1 [file DataSheet_1.zip › Table S3.PDF]

	Njällatjirelg						Reivo					
	n1	n2	m1	m2	s1	s2	n1	n2	m1	m2	s1	s2
**Ascomycota**												
Dothideomycetes												
Pleosporales												
Incertae sedis	6,85	4,59	8,00	0,41	1,36	0,11	0,88	0,52	0,91	0,93	1,09	2,94
Venturiaceae												
Venturia sp.	0,01	0,00	0,09	0,00	0,00	0,00	6,03	0,16	16,07	5,06	13,10	1,13
Other Venturiaceae	0,02	0,01	0,01	0,01	0,00	0,00	0,63	2,51	3,28	9,85	3,82	1,55
Other Pleosporales	0,21	0,11	0,45	0,03	0,06	0,00	0,26	0,38	0,10	0,25	0,28	0,22
Incertae sedis												
Myxotrichaceae												
Oidiodendron sp.	0,10	0,05	0,35	0,02	7,33	0,95	0,00	0,03	0,01	0,10	0,08	0,29
Other Myxotrichaceae	0,01	0,00	0,01	0,00	0,19	0,11	0,00	0,00	0,01	0,00	0,01	0,01
Other *Incertae sedis*	0,53	0,08	0,09	0,22	0,03	0,02	0,03	0,09	0,01	0,01	0,00	0,01
Other Dothideomycetes	2,35	1,75	1,61	1,28	0,62	0,06	2,51	1,68	3,14	2,81	1,49	3,00
u/c Dothideomycetes	0,11	0,03	0,02	0,03	0,00	0,01	2,64	0,44	1,45	0,87	0,44	1,25
Eurotiomycetes												
Chaetothyriales												
Herpotrichiellaceae												
Cladophialophora sp.	0,01	0,00	0,27	0,21	0,86	0,60	7,75	1,34	14,09	1,12	28,68	2,62
Other Herpotrichiellaceae	0,01	0,00	0,21	0,00	0,03	0,02	1,36	0,33	1,14	0,59	0,77	1,09
Other Chaetothyriales	0,01	0,00	0,06	0,01	0,04	0,01	1,10	0,30	0,50	0,30	0,31	0,34
Eurotiales												
Trichocomaceae												
Penicillium sp.	1,64	0,07	5,97	1,00	18,11	30,40	0,13	0,03	0,20	0,05	0,52	0,20
Other Trichocomaceae	0,00	0,02	0,00	0,00	0,01	0,00	0,01	0,00	0,00	0,00	0,00	0,01
Other Eurotiomycetes	0,00	0,00	0,00	0,00	0,00	0,00	0,00	0,05	0,01	0,04	0,00	0,01
u/c Eurotiomycetes	0,06	0,02	0,60	0,26	0,52	0,28	0,16	0,01	0,15	0,03	0,03	0,02
Leotiomycetes												
Helotiales												
Dermateaceae												
Incertae sedis												
Cadophora sp.	0,17	0,00	0,02	0,02	0,03	0,00	8,52	23,44	8,86	20,42	4,86	13,48
Cystodendron sp.	20,41	0,04	2,73	0,34	5,10	1,32	0,28	0,09	0,17	0,08	0,57	0,17
Other *Incertae sedis*	0,05	0,02	0,86	0,12	1,57	1,08	2,03	1,17	1,09	1,08	1,13	1,29
Hyaloscyphaceae												
Hyaloscypha sp.	0,00	0,00	0,00	0,00	0,00	0,00	0,00	0,00	0,02	2,38	0,03	18,51
Other Hyaloscyphaceae	0,76	0,44	3,84	1,56	1,63	0,80	0,19	0,17	0,22	0,21	0,22	0,12
Phacidiaceae												
Phacidium lacerum	10,87	64,50	14,33	61,32	3,99	7,16	0,74	0,38	0,57	1,55	0,09	0,23
Sclerotiniaceae	3,23	0,03	13,95	0,26	0,30	0,46	0,00	0,00	0,01	0,01	0,00	0,03
Other Helotiales	4,16	0,27	4,30	0,73	1,58	0,32	7,21	5,51	5,65	3,02	2,70	4,58
Other Letiomycetes	0,91	1,07	1,03	0,11	0,86	0,64	1,57	1,76	2,17	2,67	5,51	5,96
u/c Letiomycetes	3,88	0,53	6,67	0,87	3,09	0,92	16,80	14,37	17,29	9,06	13,43	5,45
Sordariomycetes												
Hypocreales												
Hypocreaceae												
Hypocrea sp.	1,70	0,77	1,01	4,13	0,91	10,38	0,00	0,01	0,00	0,01	0,00	0,02
Other Hypocreaceae	0,04	0,00	0,19	0,05	0,17	0,60	0,05	0,00	0,00	0,01	0,01	0,07
Other Hypocreales	0,05	0,01	0,08	0,01	0,39	0,27	0,08	0,06	0,02	0,02	0,17	0,15
Other Sordariomycetes	1,87	0,60	1,10	3,40	1,09	1,05	0,54	1,28	0,82	1,01	0,09	0,13
u/c Sordariomycetes	0,00	0,00	0,00	0,00	0,00	0,02	0,01	0,00	0,03	0,00	0,01	0,03
u/c Ascomycota	6,29	12,47	16,13	6,80	2,23	0,75	7,78	17,27	4,40	2,21	1,40	2,68
Other Ascomycota	0,39	0,08	0,34	0,13	0,26	0,00	1,56	2,32	1,67	2,30	0,82	1,70
**Basidiomycota**												
Agaricomycetes												
Agaricales												
Tricholomataceae												
Cantharellula umbonata	0,01	0,01	0,05	0,01	17,62	32,80	0,01	0,00	0,01	0,00	0,00	0,00
Other Tricholomataceae	0,00	0,00	0,21	0,00	0,03	0,00	0,00	0,00	0,00	0,00	0,00	0,00
Cantharellales												
Clavulinaceae												
Clavulina sp.	0,03	0,00	0,11	0,00	0,00	0,00	0,06	0,07	3,59	4,70	8,81	3,42
Other Cantharellales	0,00	0,00	0,01	0,00	0,00	0,00	0,00	9,45	0,01	0,52	0,10	0,77
u/c Agaricomycetes	0,01	0,03	0,12	0,08	0,01	0,02	0,37	0,21	1,16	17,07	4,68	11,95
other Agaricomycetes	0,08	0,10	0,07	0,04	1,38	0,66	0,31	0,61	0,48	0,84	1,22	0,74
Microbotryomycetes												
Sporidiobolales												
Incertae sedis												
Rhodotorula sp.	3,23	3,85	2,55	0,72	0,12	0,10	22,77	10,88	6,49	6,08	0,31	2,33
Other *Incertae sedis*	0,10	0,07	0,14	0,05	0,05	0,00	2,55	0,41	0,95	0,26	0,10	0,12
Other Sporidiobolales	0,03	0,09	0,10	0,05	0,01	0,01	0,15	0,22	0,01	0,16	0,00	0,01
Other Microbotryomycetes	0,00	0,01	0,00	0,00	0,10	0,00	0,05	0,03	0,01	0,01	0,01	0,04
u/c Microbotryomycetes	0,01	0,01	0,03	0,00	0,00	0,00	0,02	0,03	0,07	0,03	0,00	0,01
Tremellomycetes												
Cystofilobasidiales												
Cystofilobasidiaceae												
Cystofilobasidium capitatum	13,67	0,10	0,80	0,85	0,81	0,01	0,00	0,00	0,00	0,01	0,00	0,00
Other Cystofilobasidiaceae	0,59	2,28	0,93	0,10	0,00	0,00	0,08	0,08	0,10	0,09	0,08	0,10
Other Cystofilobasidiales	0,39	0,39	0,62	0,17	0,06	0,20	0,04	0,06	0,09	0,02	0,03	0,03
Filobasidiales												
Filobasidiaceae												
Cryptococcus sp.	0,58	0,39	0,19	10,02	0,13	0,15	0,57	0,06	0,16	0,04	0,13	0,08
Other Filobasidiaceae												
Tremellales												
Incertae sedis												
Cryptococcus victoriae	8,34	4,13	3,06	2,65	0,17	0,23	0,15	0,09	0,01	0,03	0,01	0,01
Other *Incertae sedis*	0,18	0,25	0,19	0,47	0,06	0,00	0,19	0,10	0,05	0,07	0,05	0,06
Other Tremellales	0,00	0,00	0,00	0,00	0,00	0,00	0,00	0,00	0,00	0,00	0,02	0,00
u/c Tremellomycetes	0,23	0,15	0,13	0,55	0,45	0,02	0,18	0,21	0,11	0,09	0,06	0,20
Other Tremellomycetes	1,45	0,06	0,73	0,12	0,06	0,01	0,04	0,19	0,02	0,02	0,01	0,01
u/c Basidiomycota	0,44	0,12	0,19	0,25	0,08	0,02	0,49	0,61	0,41	0,27	0,20	0,33
Other Basidiomycota	0,01	0,00	0,02	0,02	0,00	0,00	0,11	0,13	0,09	0,06	0,02	0,04
**Zygomycota**												
Mortierellales												
Mortierellaceae												
Mortierella sp.	2,79	0,04	3,40	0,19	24,46	6,33	0,06	0,03	0,13	0,14	0,41	4,83
Other Zygomycota	0,28	0,01	0,35	0,08	1,52	1,06	0,00	0,00	0,00	0,00	0,00	0,00
Other fungal phyla	0,86	0,34	1,71	0,26	0,52	0,04	0,96	0,84	2,01	1,45	2,11	5,64
Unclassified Fungi	0,00	0,00	0,00	0,00	0,00	0,01	0,00	0,00	0,00	0,01	0,01	0,00

Table S3 Fungal community composition of the most abundant taxa with depth across the different moss tissue type: 'light green' (n) corresponding to new growth tissue from the first 1 cm from the apex, 'dark green' (m) to the mature photosynthetically active segment below the new growth and 'brown/senescent tissue' (s) corresponding to the senesced segment. Samples were collected at two different sites: Njällatjirelg, an open canopy forest with high forest floor moss nitrogenase activity (N2 fixation); Reivo, a variably dense canopy forest with moderately high N2 fixation in the moss layer. Subscripted number refers the two pooled samples per site (1 and 2 from locations 1-6 and 7-12 respectively).
