# Supplementary material for: Revealing the transfer pathways of cyanobacterial-fixed N into the boreal forest through the feather-moss microbiome
Source: Front Plant Sci. 2022 Dec 9;13:1036258. doi: 10.3389/fpls.2022.1036258 (PMC9780503; doi:10.3389/fpls.2022.1036258)
Supplement: Supplementary file 1 [file DataSheet_1.zip › Table S5.PDF]

		Njällatjirelg						Reivo					
	Type	n1	n2	m1	m2	s1	s2	n1	n2	m1	m2	s1	s2
Clavulina sp.	ECM	5	0	19	0	0	0	10	12	619	809	1518	590
Oidiodendron sp.	ERM	17	9	60	4	1262	164	0	5	2	18	13	50
Cortinarius sp.	ECM	0	0	3	2	27	3	14	17	36	23	48	53
Tylospora sp.	ECM	0	0	0	1	0	18	10	4	2	0	1	3
Piloderma sp.	ECM	0	0	0	0	7	11	0	0	5	5	4	0
Chloridium sp.	ECM	0	0	0	0	1	18	0	0	0	0	0	0
Amphinema sp.	ECM	0	0	0	0	3	0	3	3	0	2	0	2
Pseudogymnoascus sp.	ERM	0	0	1	3	0	3	0	0	0	0	0	0
Byssocorticium sp.	ECM	0	0	0	0	0	0	5	1	0	1	0	0
Tomentella sp.	ECM	0	0	0	0	0	0	5	0	1	0	1	0
Lactarius sp.	ECM	0	0	0	0	0	1	0	0	1	0	1	2
Tomentellopsis sp.	ECM	0	0	0	0	0	0	1	0	1	0	0	1
Russula sp.	ECM	0	0	0	0	0	0	0	0	0	0	2	0
Entoloma sp.	ECM	0	0	0	0	0	1	0	0	0	0	0	0
Suillus sp.	ECM	0	0	0	0	0	0	0	0	1	0	0	0
Sistotrema sp.	ECM	0	0	0	0	0	0	0	0	0	0	1	0

Table S5 Mycorrhizal fungi (ECM: Ectomycorrhiza ;ERM: Ericoid Mycorrhiza) abundance with depth across the different moss tissue type: 'light green' (n) corresponding to new growth tissue from the first 1 cm from the apex, 'dark green' (m) to the mature photosynthetically active segment below the new growth and 'brown/senescent tissue' (s) corresponding to the senesced segment. Samples were collected at two different sites: Njällatjirelg, an open canopy forest with high forest floor moss nitrogenase activity (N2 fixation); Reivo, a variably dense canopy forest with moderately high N2 fixation in the moss layer. Subscripted number refers the two pooled samples per site (1 and 2 from locations 1-6 and 7-12 respectively).
