# Supplementary material for: Revealing the transfer pathways of cyanobacterial-fixed N into the boreal forest through the feather-moss microbiome
Source: Front Plant Sci. 2022 Dec 9;13:1036258. doi: 10.3389/fpls.2022.1036258 (PMC9780503; doi:10.3389/fpls.2022.1036258)
Supplement: Supplementary file 1 [file DataSheet_1.zip › Table S6.PDF]

Site	Stem position	Replicate	No. bacterial OTUs S_Obs	S_Chao1	SE	No. fungal OTUs S_Obs	S_Chao1	SE
NJA	n	1	150	273	43	259	397	39
NJA	n	2	88	162	34	135	234	38
NJA	m	1	151	209	22	321	493	43
NJA	m	2	114	183	28	190	318	43
NJA	s	1	258	428	46	329	521	47
NJA	s	2	339	570	53	221	317	27
REV	n	1	192	287	32	390	450	18
REV	n	2	196	327	44	351	464	30
REV	m	1	259	447	53	493	590	22
REV	m	2	241	364	35	429	596	38
REV	s	1	329	611	69	427	580	33
REV	s	2	354	653	69	470	686	46

Table S6 Chao1 estimates of underlying community richness, with standard errors (n = new growth tissue from the first 1 cm from the moss apex; m = mature photosynthetically active segment below the new growth segment; s = senesced segment; S_obs = observed richness in the standardised sample, S_Chao 1 = estimated richness for the sample, SE = the standard error associated with estimated richness). Samples were collected at two different sites: Njällatjirelg (NJA), an open canopy forest with high rates of forest floor moss nitrogenase activity (N2 fixation); Reivo (REV), a variably dense canopy forest with moderately high N2 fixation in the moss layer.
