# Supplementary material for: Revealing the transfer pathways of cyanobacterial-fixed N into the boreal forest through the feather-moss microbiome
Source: Front Plant Sci. 2022 Dec 9;13:1036258. doi: 10.3389/fpls.2022.1036258 (PMC9780503; doi:10.3389/fpls.2022.1036258)
Supplement: Supplementary file 1 [file Table S4.PDF › Table S4.PDF]

		Njällatjirelg						Reivo					
	Trophic Mode	n1	n2	m1	m2	s1	s2	n1	n2	m1	m2	s1	s2
Acicuseptoria	?	17	0	11	0	0	0	0	0	0	0	0	0
Archaeorhizomyces	?	0	0	0	0	0	0	4	0	56	0	10	2
Celosporium	?	0	0	0	0	0	0	0	0	3	0	0	0
Cystofilobasidium	?	2355	18	138	147	139	2	0	0	0	1	0	0
Fellomyces	?	0	0	0	0	0	0	7	0	1	0	0	0
Friedmanniomyces	?	0	0	0	0	0	0	0	0	0	2	0	0
Fulvoflamma	?	0	0	1	0	2	0	1	0	1	0	0	0
Gremmeniella	?	1	0	0	0	0	0	0	0	0	0	0	1
Guehomyces	?	11	0	0	0	0	0	0	0	0	0	0	0
Hispidoconidioma	?	3	0	0	0	0	0	24	13	19	17	3	17
Kurtzmanomyces	?	0	0	0	0	0	0	0	0	2	0	0	0
Lalaria	?	5	0	1	0	0	0	0	0	2	0	0	0
Leucosporidiella	?	0	0	0	0	18	0	0	2	0	1	0	3
Lipomyces	?	0	0	0	0	0	0	0	1	0	4	0	5
Neostagonospora	?	0	1	0	0	0	0	0	1	0	0	0	0
Phaeosclera	?	0	0	0	0	0	0	0	0	0	0	0	1
Phaeotheca	?	1	0	0	0	0	0	0	0	3	0	0	0
Rachicladosporium	?	1	0	0	0	0	0	0	0	3	1	0	2
Stylonectria	?	8	0	0	0	0	0	0	9	0	0	0	0
Troposporella	?	3	0	0	0	0	0	0	0	0	0	0	1
Udeniomyces	?	86	392	47	17	0	0	13	14	18	16	13	18
Xanthophyllomyces	?	5	0	114	0	0	0	0	0	0	0	0	0
Geomyces	PAT/SAP	0	0	0	0	4	9	0	0	0	0	0	0
Leptodontidium	PAT/SAP	5	3	27	14	255	183	201	192	127	156	149	187
Aspergillus	PAT/SAP	0	3	0	0	0	0	0	0	0	0	0	2
Cadophora	PAT/SYM	30	0	4	3	5	0	1467	4038	1527	3517	838	2322
Cryptococcus	PAT/SAP	100	67	33	1727	22	25	98	10	28	7	23	13
Cryptococcus	PAT/SAP	1466	754	557	535	40	40	46	29	9	13	9	10
Phialocephala	PAT/SYM	0	0	0	0	0	1	14	0	3	1	1	2
Alternaria	PAT	0	0	0	0	0	0	0	0	0	0	1	0
Ascochyta	PAT	7	0	0	0	1	0	0	0	0	0	0	0
Aureobasidium	PAT	55	191	18	52	0	1	1	0	0	0	0	0
Beauveria	PAT	0	0	0	0	0	0	0	0	0	1	1	1
Botrytis	PAT	0	0	78	0	0	74	0	0	1	0	0	2
Calcarisporium	PAT	0	0	0	0	1	0	0	0	0	0	0	0
Capronia	PAT	0	0	0	0	0	0	0	2	5	0	4	3
Ceratobasidium	PAT	0	0	0	0	0	0	0	0	0	0	9	0
Chalara	PAT	23	10	33	14	4	0	5	119	15	23	7	24
Chalastospora	PAT	0	0	0	1	0	0	0	0	0	0	0	0
Cladosporium	PAT	5	0	0	0	0	0	0	0	0	0	0	0
Coccomyces	PAT	0	0	0	0	0	0	0	0	0	2	0	42
Collophora	PAT	11	0	15	0	40	0	1	1	2	0	0	0
Cylindrosympodium	PAT	0	0	0	0	0	0	4	0	3	0	0	0
Cystodendron	PAT	3516	7	470	58	879	227	49	15	29	13	99	30
Cytospora	PAT	0	0	0	3	0	0	0	0	0	0	0	0
Devriesia	PAT	1	0	0	0	0	0	0	0	0	5	0	1
Dothiorella	PAT	0	2	0	0	0	0	8	3	3	3	2	5
Exobasidium	PAT	1	0	3	1	0	0	18	21	6	6	0	6
Haptocillium	PAT	0	0	0	0	0	1	0	0	0	1	0	3
Hyphodiscus	PAT	0	0	1	0	4	0	57	3	21	12	5	11
Hypocrea	PAT	292	133	174	712	156	1789	0	1	0	2	0	4
Hypomyces	PAT	0	0	0	0	0	0	6	0	0	0	0	0
Lecanicillium	PAT	0	0	0	0	3	12	0	0	0	0	0	0
Lecythophora	PAT	4	2	56	21	174	153	7	0	0	0	0	1
Leiosphaerella	PAT	0	0	0	0	0	0	0	0	0	2	0	0
Leptosphaeria	PAT	4	4	2	5	0	0	0	0	0	10	0	10
Lophodermium	PAT	0	0	0	0	0	0	72	0	50	5	2	1
Meliniomyces	PAT	0	0	5	0	40	0	13	6	14	2	270	345
Microdochium	PAT	0	11	0	1	0	0	0	0	0	0	0	0
Mollisia	PAT	1	0	0	0	0	0	0	2	0	2	0	0
Mucor	PAT	27	0	41	0	11	22	0	0	0	0	0	0
Ophiostoma	PAT	0	0	0	0	0	1	0	0	0	0	0	0
Paecilomyces	PAT	0	0	0	0	0	0	1	0	0	0	0	0
Penidiella	PAT	0	0	1	0	0	0	0	0	0	0	0	1
Phacidium	PAT	1873	11112	2469	10563	687	1233	128	65	98	267	15	39
Phoma	PAT	2	0	0	0	0	0	0	0	1	0	0	0
Physalospora	PAT	5	0	0	3	0	0	0	0	0	0	0	0
Pochonia	PAT	0	0	3	1	28	14	0	0	1	0	19	9
Powellomyces	PAT	0	0	0	0	0	0	9	0	11	1	7	8
Pyrenopeziza	PAT	0	0	1	1	0	0	0	0	0	0	0	0
Resinicium	PAT	0	0	0	0	0	0	0	1	2	0	0	2
Rhizosphaera	PAT	0	2	0	0	0	0	0	0	0	0	0	0
Sarea	PAT	0	0	0	0	0	0	4	5	2	4	3	3
Scleroconidioma	PAT	3	14	2	35	1	0	11	0	2	1	0	0
Sclerotinia	PAT	0	5	0	0	0	0	0	0	0	0	0	0
Simplicillium	PAT	0	0	2	0	8	0	8	0	0	0	2	5
Sirococcus	PAT	1	0	0	0	0	0	0	0	0	0	0	0
Sporobolomyces	PAT	0	1	1	0	3	0	294	32	105	16	7	6
Syzygospora	PAT	48	7	47	11	7	1	5	5	12	1	5	4
Taphrina	PAT	1	0	0	0	0	0	4	2	3	3	0	0
Torula	PAT	0	0	0	1	0	0	0	0	0	0	0	0
Triposporium	PAT	0	0	0	0	0	0	4	0	0	0	0	0
Ustilago	PAT	0	0	1	0	0	0	0	0	0	0	0	0
Venturia	PAT	1	0	15	0	0	0	1039	28	2769	871	2256	195
Tetracladium	SAP	0	0	1	0	0	0	0	0	0	0	0	0
Acanthophysium	SAP	1	0	0	0	0	0	0	0	0	0	0	0
Arachnopeziza	SAP	0	0	0	0	0	0	0	0	2	0	1	0
Arrhenia	SAP	0	0	36	0	5	0	0	0	0	0	0	0
Arthrinium	SAP	0	0	0	0	5	1	0	0	0	0	0	0
Basidiodendron	SAP	0	0	0	0	1	0	0	0	0	0	0	0
Bensingtonia	SAP	0	0	0	0	0	0	0	0	0	0	3	0
Cantharellula	SAP	1	1	8	2	3036	5651	1	0	1	0	0	0
Capnobotryella	SAP	0	0	0	0	0	0	18	4	7	6	1	6
Ceramothyrium	SAP	0	0	1	0	2	0	0	3	0	2	0	1
Chaetosphaeria	SAP	0	0	0	0	0	0	0	0	0	0	1	0
Cistella	SAP	128	75	660	268	138	119	27	28	18	24	35	19
Cladophialophora	SAP	2	0	46	37	149	104	1335	231	2427	193	4941	451
Claussenomyces	SAP	2	0	1	1	9	0	0	39	0	1	0	3
Clavatospora	SAP	0	0	0	0	0	0	0	2	0	0	0	0
Clavulinopsis	SAP	0	0	0	0	6	1	0	0	0	0	0	0
Coniochaeta	SAP	0	0	0	56	0	0	0	0	0	1	1	0
Coniophora	SAP	0	0	1	0	0	0	0	0	1	0	0	0
Crocicreas	SAP	0	0	0	0	15	2	5	0	1	1	0	12
Davidiella	SAP	203	50	139	26	7	0	22	7	15	6	8	4
Dioszegia	SAP	0	1	0	2	0	0	0	0	0	0	0	2
Endoconidioma	SAP	0	0	0	4	1	0	0	0	0	0	0	0
Exophiala	SAP	0	0	0	0	1	0	15	0	55	0	4	1
Fomes	SAP	1	0	0	0	2	0	5	49	5	71	2	20
Galerina	SAP	0	0	0	0	57	37	0	1	0	3	2	3
Gymnopus	SAP	0	0	1	0	9	0	0	0	0	0	0	0
Helicoma	SAP	0	0	0	0	0	0	0	0	2	0	0	0
Heyderia	SAP	0	0	0	0	0	0	1	0	2	7	0	1
Hyaloscypha	SAP	0	0	0	0	0	0	0	0	3	410	5	3188
Hymenoscyphus	SAP	0	0	1	0	0	0	0	6	1	2	17	0
Hyphodontia	SAP	0	0	0	0	0	0	0	0	0	1	0	0
Hyphodontiella	SAP	0	0	0	0	0	0	0	0	0	0	0	4
Hypholoma	SAP	1	0	0	3	2	0	1	0	2	5	0	1
Hypochnicium	SAP	0	0	0	0	0	0	0	2	4	0	0	1
Hypoxylon	SAP	1	0	0	0	0	0	0	0	0	0	0	0
Junghuhnia	SAP	0	0	0	0	0	0	0	0	0	1	0	1
Kabatiella	SAP	1	0	14	0	61	1	0	0	0	0	0	0
Kabatina	SAP	1	0	4	0	0	0	0	0	0	0	0	0
Kockovaella	SAP	0	0	0	0	0	0	0	1	0	0	0	0
Lachnellula	SAP	1	0	0	0	0	0	3	2	13	13	2	0
Lachnum	SAP	0	0	1	0	0	0	0	0	0	0	0	0
Leucogyrophana	SAP	0	0	0	0	0	5	0	0	0	0	0	0
Lophium	SAP	1	0	0	0	0	0	5	17	4	21	3	3
Lycoperdon	SAP	0	0	0	0	1	0	0	0	0	0	0	0
Mortierella	SAP	480	7	585	32	4214	1090	11	5	22	24	70	832
Mycena	SAP	0	0	2	0	14	4	0	6	0	0	20	3
Penicillium	SAP	283	12	1028	173	3120	5237	23	6	34	9	90	35
Peniophora	SAP	0	0	1	0	0	0	0	0	0	0	0	0
Phaeococcomyces	SAP	1	0	1	0	0	0	2	11	12	0	2	0
Phaeomoniella	SAP	0	0	0	0	0	0	7	7	2	1	2	0
Phaeosphaeria	SAP	0	0	0	0	0	0	1	0	0	0	0	0
Phaeothecoidea	SAP	0	0	0	0	0	0	0	0	5	0	0	0
Phialea	SAP	0	0	0	0	0	0	0	127	0	16	3	0
Phlebiella	SAP	0	0	0	0	0	0	0	0	0	0	1	0
Pholiota	SAP	1	0	0	0	5	2	0	0	0	0	0	0
Preussia	SAP	0	0	0	0	0	0	18	32	4	0	35	0
Psathyrella	SAP	0	1	0	0	0	0	0	0	0	0	0	0
Pseudoplectania	SAP	0	0	0	0	1	0	0	0	1	1	3	1
Rhodotorula	SAP	557	663	439	124	20	17	3922	1875	1118	1048	53	402
Sagenomella	SAP	0	0	0	0	1	0	0	0	0	0	0	0
Scleropezicula	SAP	0	0	117	0	11	0	14	5	21	8	24	18
Sclerostagonospora	SAP	0	0	1	0	0	0	0	0	0	0	0	0
Sistotremastrum	SAP	0	0	0	0	0	0	0	0	1	0	0	0
Sporormiella	SAP	0	0	0	0	1	0	0	5	0	0	1	0
Stropharia	SAP	0	0	0	0	0	0	0	0	0	1	1	0
Sympodiella	SAP	0	0	0	0	0	0	95	0	32	0	25	3
Trechispora	SAP	0	0	0	0	0	0	0	1	1	3	0	0
Tubulicrinis	SAP	0	0	0	0	0	0	0	0	0	1	0	0
Umbelopsis	SAP	22	1	19	13	250	161	0	0	0	0	0	0
Xenasmatella	SAP	0	0	0	0	0	0	0	0	4	2	4	1
Xenopolyscytalum	SAP	1	0	1	0	0	0	77	2	17	10	16	2
Zalerion	SAP	0	0	0	0	0	0	0	0	1	1	1	0
Russula	SYM	0	0	0	0	0	0	0	0	0	0	2	0
Amphinema	SYM	0	0	0	0	3	0	3	3	0	2	0	2
Byssocorticium	SYM	0	0	0	0	0	0	5	1	0	1	0	0
Chloridium	SYM	0	0	0	0	1	18	0	0	0	0	0	0
Clavulina	SYM	5	0	19	0	0	0	10	12	619	809	1518	590
Cortinarius	SYM	0	0	3	2	27	3	14	17	36	23	48	53
Entoloma	SYM	0	0	0	0	0	1	0	0	0	0	0	0
Oidiodendron	SYM	17	9	60	4	1262	164	0	5	2	18	13	50
Lactarius	SYM	0	0	0	0	0	1	0	0	1	0	1	2
Piloderma	SYM	0	0	0	0	7	11	0	0	5	5	4	0
Pseudogymnoascus	SYM	0	0	1	3	0	3	0	0	0	0	0	0
Sistotrema	SYM	0	0	0	0	0	0	0	0	0	0	1	0
Suillus	SYM	0	0	0	0	0	0	0	0	1	0	0	0
Tomentella	SYM	0	0	0	0	0	0	5	0	1	0	1	0
Tomentellopsis	SYM	0	0	0	0	0	0	1	0	1	0	0	1
Tylospora	SYM	0	0	0	1	0	18	10	4	2	0	1	3
Aspicilia	SYM	0	0	0	0	0	0	0	0	12	0	9	0
Bryoria	SYM	0	0	0	0	0	0	0	39	7	20	0	0
Calicium	SYM	0	0	0	0	0	0	4	2	2	5	1	6
Cetraria	SYM	1	0	0	0	0	0	1	0	2	1	0	1
Cladonia	SYM	6	0	6	0	3	0	22	14	20	12	6	23
Elixia	SYM	0	0	0	0	0	0	0	0	4	0	0	0
Hypocenomyce	SYM	0	0	0	0	0	0	0	0	0	3	0	4
Hypogymnia	SYM	0	0	0	0	0	0	0	0	1	3	0	0
Lecanora	SYM	0	0	0	0	0	0	0	0	0	2	0	1
Micarea	SYM	0	0	0	0	0	0	0	0	0	2	0	12
Mycocalicium	SYM	0	0	0	0	0	0	0	9	1	7	0	2
Ochrolechia	SYM	0	0	0	0	0	0	0	0	16	3	0	0
Parmelia	SYM	0	0	0	0	0	0	2	2	0	1	0	0
Parmeliopsis	SYM	0	0	0	0	0	0	81	26	16	50	1	48
Phylliscum	SYM	0	0	0	0	0	0	0	0	0	2	0	0
Placynthiella	SYM	0	0	0	0	0	0	8	3	0	28	6	9
Platismatia	SYM	0	0	0	0	0	0	0	0	8	1	0	0
Trapeliopsis	SYM	0	0	1	0	0	0	7	12	4	46	57	79
		7	0	7	0	3	0	125	107	93	186	80	185

Table S4 Abundance and putative trophic mode (SAP = saprotroph; PAT = pathotroph; SYM = symbiotroph) of fungal taxa that could be resolved to genus level (190 in total) with depth across the different moss tissue type: 'light green' (n) corresponding to new growth tissue from the first 1 cm from the apex, 'dark green' (m) to the mature photosynthetically active segment below the new growth and 'brown/senescent tissue' (s) corresponding with the senesced segment. Samples were collected at two different sites: Njällatjirelg, an open canopy forest with high forest floor moss nitrogenase activity (N2 fixation); Reivo, a variably dense canopy forest with moderately high N2 fixation in the moss layer. Subscripted number refers the two pooled samples per site (1 and 2 from locations 1-6 and 7-12 respectively).
